# Parallel mapping with site-directed hydroxyl radicals and micrococcal nuclease reveals structural features of positioned nucleosomes *in vivo*

**DOI:** 10.1371/journal.pone.0186974

**Published:** 2017-10-26

**Authors:** Tomohiro Fuse, Koji Katsumata, Koya Morohoshi, Yukio Mukai, Yuichi Ichikawa, Hitoshi Kurumizaka, Akio Yanagida, Takeshi Urano, Hiroaki Kato, Mitsuhiro Shimizu

**Affiliations:** 1 Department of Chemistry, Graduate School of Science and Engineering, Program in Chemistry and Life Science, School of Science and Engineering, Meisei University, Hino, Tokyo, Japan; 2 Department of Bioscience, Faculty of Bioscience, Nagahama Institute of Bio-Science and Technology, Nagahama, Shiga, Japan; 3 Graduate School of Advanced Science and Engineering/RISE/IMSB, Waseda University, Shinjuku-ku, Tokyo, Japan; 4 School of Pharmacy, Tokyo University of Pharmacy and Life Science, Hachioji, Tokyo, Japan; 5 Department of Biochemistry, Shimane University School of Medicine, Izumo, Shimane, Japan; National Cancer Institute, UNITED STATES

## Abstract

Micrococcal nuclease (MNase) has been widely used for analyses of nucleosome locations in many organisms. However, due to its sequence preference, the interpretations of the positions and occupancies of nucleosomes using MNase have remained controversial. Next-generation sequencing (NGS) has also been utilized for analyses of MNase-digests, but some technical biases are commonly present in the NGS experiments. Here, we established a gel-based method to map nucleosome positions in *Saccharomyces cerevisiae*, using isolated nuclei as the substrate for the histone H4 S47C-site-directed chemical cleavage in parallel with MNase digestion. The parallel mapping allowed us to compare the chemically and enzymatically cleaved sites by indirect end-labeling and primer extension mapping, and thus we could determine the nucleosome positions and the sizes of the nucleosome-free regions (or nucleosome-depleted regions) more accurately, as compared to nucleosome mapping by MNase alone. The analysis also revealed that the structural features of the nucleosomes flanked by the nucleosome-free region were different from those within regularly arrayed nucleosomes, showing that the structures and dynamics of individual nucleosomes strongly depend on their locations. Moreover, we demonstrated that the parallel mapping results were generally consistent with the previous genome-wide chemical mapping and MNase-Seq results. Thus, the gel-based parallel mapping will be useful for the analysis of a specific locus under various conditions.

## Introduction

In eukaryotic chromatin, the nucleosome is the basic structural and functional unit, consisting of 145–147 bp DNA wrapped around the histone octamer, composed of two molecules each of histones H2A, H2B, H3 and H4 [1,2]. The locations of nucleosomes in genomes have been studied in many organisms, and it is widely accepted that nucleosome positioning is an important mechanism to control gene expression as well as epigenetic regulation [3–6]. Recent studies have revealed the existence of non-canonical nucleosomes *in vivo* and *in vitro*, such as fragile nucleosomes [7–10], subnucleosomes [9,11], asymmetric nucleosomes [12,13], prenucleosomes [14], hexasomes [15–18], hemisomes [19–21], proto-chromatosomes [22] and overlapping dinucleosomes [23–25]. However, the structural features and functional consequences of these non-canonical nucleosomes *in vivo* remain to be elucidated. Thus, the development of a method to analyze nucleosomes *in vivo* is eagerly awaited.

To determine the locations of nucleosomes, micrococcal nuclease (MNase) is often used, since the linker DNA regions between two nucleosomes are preferentially cut by MNase, whereas the DNA regions within nucleosomes tend to be protected. Hence, by comparing the digestion pattern in chromatin with that in the naked DNA by a gel electrophoretic analysis, the cleaved and protected regions are assigned as the linker and nucleosomal DNA regions, respectively. In addition, mono-nucleosome-size DNA fragments isolated from MNase digestions of chromatin in nuclei have been analyzed by massive parallel sequencing or high-density DNA microarrays, to determine the nucleosome positions in the whole genome. However, MNase cleaves DNA in a sequence-dependent manner; *i*.*e*., the 5' side of A•T base pairs is preferentially cut, while certain sequences such as G+C-rich regions are not digested well, even in naked DNA [26,27]. In addition to the endonuclease activity, MNase possesses an exonuclease activity [28–30] that affects the trimming of the ends of nucleosomal DNA after cleavage of the linker DNA regions between the nucleosomes [29,30]. Thus, if a nucleosome has a neighboring linker sequence that is poorly digested by MNase, its precise position would be difficult to deduce. In fact, the mono-nucleosome-size DNA fragments generated by MNase reportedly have a sequence-dependent bias, which might affect the interpretation of nucleosome positions and occupancies [28–32]. An attempt to assess the potential biases caused by MNase led to the conclusion that this enzyme does not substantially bias the mapping results, at least when reconstituted chromatin was used as the substrate [33]. Thus, whether the enzymatic biases strongly affect the mapping results remains controversial. To interpret MNase mapping results more accurately, an MNase titration assay combined with chromatin immunoprecipitation [31], MPE-seq [the digestion of nuclei with MPE-Fe(II) followed by massive parallel sequencing] [9], and combined MNase/exonuclease III digestion mapping [22,34] have been reported recently.

In comparison with MNase, hydroxyl radicals have minimal sequence preference for cleaving DNA, since they attack the sugar moiety in the DNA backbone [35]. Thus, hydroxyl radicals, which are typically generated by the Fenton reaction with Fe^2+^ and H_2_O_2_, have been widely used for DNA footprinting studies to analyze the specific binding sites for proteins and the helical periodicity of DNA in the nucleosome [36–38]. Site-directed hydroxyl radical cleavages have also been successfully applied to detect the locations of histone H4 with the S47C mutation [39], H2B with the T87C mutation [40], and the linker histone H1^0^ with the G101C mutation [41] *in vitro*. In these methods, a target amino acid residue was replaced with a cysteine residue in these histones, to conjugate ferrous-ion-chelating reagents. Brogaard *et al*. developed a genome-wide chemical mapping approach that directly determines the nucleosome center positions with single-base-pair resolution *in vivo* [42,43]. They purified the DNA fragments from the H4 S47C-dependent chemical cleavage reaction, which occurs around the center of the nucleosome where the S47C residue is located. Then, they size-selected the fragments to the mono-nucleosome-size, which is comparable to the distance between the cleavage sites on two juxtaposed nucleosomes, and analyzed them by deep sequencing. Similarly, the chemical maps of nucleosomes in *Schizosaccharomyces pombe* [44] and mouse embryonic stem cells [45] have been successfully reported, although few studies have focused on the validation of the chemical mapping of nucleosomes *in vivo*.

Next-generation sequencing (NGS) analyses of mono-nucleosome-size fragments, obtained by digestions with MNase or hydroxyl radicals, are a powerful technique to obtain an overview of the genome-wide distribution of nucleosomes. However, the size-selected DNA fragments may have a bias in their populations and some DNA fragments from certain nucleosomes might be missed, since nucleosomes are not always cleaved uniformly by MNase or chemical cleavages. The local chromatin structures *in vivo* vary in different chromosome locations and in responses to biological processes, such as DNA transcription, replication, repair and recombination. In fact, variations reportedly exist in the susceptibility of individual nucleosomes to MNase [7–10,46,47]; *i*.*e*., certain nucleosomes are more easily digested with MNase, supporting the idea that the structural features and the stability of nucleosomes are not uniform, and instead dynamically change within the genome. In addition, it has been pointed out that some technical biases are commonly present in the NGS experiments, since each step of NGS library preparation, such as end-repair of DNA fragments, adapter ligation, size selection of DNA fragments, and PCR amplification, is a potential source of bias [48,49].

Here, we established a conventional gel-based method for nucleosome mapping *in vivo*, by indirect end-labeling and primer extension mapping using the H4 S47C-dependent site-directed chemical cleavage, combined in parallel with MNase digestion. So far, we have been studying nucleosome positioning in yeast minichromosomes as well as in the genome, using isolated nuclei limitedly digested by MNase to different extents [50–55]. Using the isolated nuclei as the substrate for the chemical cleavage as well as the MNase digestion, followed by indirect end-labeling or primer extension mapping, we were able to map the linker DNA regions and the centers of nucleosomes. We examined the chromatin structure of the TRP1ARS1 minichromosome and the same DNA region of the *TRP1* locus in the yeast chromosome IV. We show that the positions of the nucleosomes were mapped more accurately by this gel-based parallel mapping, as compared to MNase mapping alone.

## Materials and methods

### Yeast strains and plasmids

The yeast strains used in this study are listed in Table A in S1 File. The Mat-alpha-YDR007W and Mat-alpha-YBR009C strains, which were derived from BY4742, were purchased from Open Biosystems. The H4 S47C strain (MYA-4902), which was constructed by Brogaard *et al*. [42,43], was purchased from the ATCC. The *trp1*::*KanMX* mutation was introduced to MYA-4902, to form the strain MHS3001. To obtain the *MAT*α H4 S47C strain with the genetic background of BY4742, MHS3001 and Mat-alpha-YBR009C were crossed, and the diploid cells thus obtained were sporulated, dissected to haploid cells and screened for the MHS3002 strain, which carried the *hhf1*::*S47C* and *hhf2*::*URA3* mutations. We also introduced *hhf2*::*KanMX* and *hhf1*::*S47C* into the FY23 and FY24 strains, respectively, thus constructing the MHS3003 and 3004 strains. MHS3003 and 3004 were crossed, and dissected to haploid cells to screen for the isogenic MHS3005 and 3006 strains. The H4 S47C mutation in these strains was confirmed by DNA sequencing. Culture conditions, media, strain construction and transformations followed standard recipes and protocols, as described [56].

The plasmid TALS-pBR∆RI [54] was digested with *Eco*RI and self-ligated, forming TRP1ARS1-pBR∆RI. The TRP1ARS1-pBR∆RI plasmid was digested with *Hin*dIII, to eliminate the pBR322 portion. The TRP1ARS1 fragment was recovered, ligated, and introduced into the yeast strains. Culture conditions, media and transformations followed standard recipes and protocols, as described [56].

### Isolation of yeast nuclei

Yeast nuclei were isolated as described previously [55,57,58]. Figure A in S1 File shows the scheme of the parallel mapping procedures, including the isolation of yeast nuclei. Yeast cells harboring the TRP1ARS1 plasmid were cultured in 1L of SC-Trp medium until the OD_600_ reached 1.0–1.5. Then, sodium azide and phenylmethanesulfonyl fluoride (PMSF) were added to the medium, at final concentrations of 0.13% and 0.5 mM, respectively. The cells were harvested, washed once with S-buffer (1.4 M D-sorbitol, 40 mM HEPES (pH 7.5), 0.5 mM MgCl_2_, 0.13% NaN_3_, 1 mM PMSF), resuspended in S-buffer, and treated with Zymolyase for conversion to spheroplasts. The spheroplasts were homogenized, and subjected to step gradient centrifugation and subsequent differential centrifugation to isolate nuclei.

### Site-directed chemical cleavages of chromatin in isolated nuclei

The nuclei pellet from 1L culture was resuspended in 4 ml of labeling buffer (1 M sorbitol, 50 mM NaCl, 10 mM Tris-HCl, pH 7.5, 5 mM MgCl_2_, 0.5 mM spermidine, 0.15 mM spermine). For labeling, N-(1,10 phenanthroline-5-yl) iodoacetamide (Biotium cat# 92015) was dissolved in dimethylsulfoxide to a concentration of 0.7 mM, and added to the nuclei suspension to a final concentration of 0.14 mM of the labeling reagent and a 20% total volume of dimethylsulfoxide. The labeling reaction was incubated with rotation at 4°C for 1 hr in the dark. After the reaction, the nuclei were recovered by centrifugation at 4,000xg for 10 min, and resuspended in 3 ml of labeling buffer. The nuclei were washed with labeling buffer four times, and resuspended in 3 ml of mapping buffer (1 M sorbitol, 2.5 mM NaCl, 50 mM Tris-HCl, pH 7.5, 5 mM MgCl_2_, 0.5 mM spermidine, 0.15 mM spermine). CuCl_2_ was added to the nuclei suspension to a final concentration of 0.01 mM, and the suspension was incubated for 5 min at room temperature. After centrifugation at 4,000xg for 10 min, the nuclei were washed three times with 3 ml of mapping buffer, and resuspended in 1.625 ml of mapping buffer. 3-Mercaptopropionic acid was added to a final concentration of 5.9 mM, and the suspension was incubated for 5 min at room temperature. The chemical reaction was initiated by the addition of 0.4 M hydrogen peroxide to a final concentration of 6 mM, and aliquots of the reaction mixture (330 μl) were transferred to a new tube containing 2μl of 0.5 M neocuproine (Sigma), which resulted in a final concentration of 3 mM, to quench the reactions at 5, 10, 15 and 20 min (see Figure A in S1 File). The nuclei were pelleted and resuspended in 200μl of mapping buffer, and the DNA was purified from the nuclei as described previously [59]. The chemical cleavage in the genomic DNA was confirmed by agarose gel electrophoresis.

### MNase digestion of chromatin in isolated nuclei

The MNase digestion of the isolated nuclei was described previously [51–55]. The nuclei pellet was resuspended in 2 ml of digestion buffer [10 mM HEPES (pH 7.5), 0.5 mM MgCl_2_, 0.05 mM CaCl_2_, 1 mM PMSF]. The suspended nuclei were divided into 200 μl portions and digested at 37°C for 10 min, using successive 2-fold serial dilutions of MNase (10 to 0.625 units/ml) (see Figure A in S1 File). The DNA was purified as described previously [59]. The purified DNA was digested with 10- to 60-fold lower concentrations of nucleases, to provide naked DNA controls. The digestion of the genomic DNA was confirmed by agarose gel electrophoresis.

### Mapping of cleavage sites

Chemical and MNase cleavage sites were analyzed by indirect end-label mapping or by primer extension mapping, as described previously [51–55]. The *Eco*RV-*Hin*dIII and *Nhe*I-*Stu*I (positions 388 to 619 and 830 to 1041, respectively) DNA fragments in TRP1ARS1, which were prepared by PCR, were radioactively labeled using a BcaBest Labeling kit (TAKARA) with random primers, and used as the probes for indirect end-labeling. The primers used for primer extension mapping are as follows: NII_top_primer (sequence of the top strand 919 to 954), 5'-TTG ATT GTA CAG GAA AAT ATA CAT CGC AGG GGG TTG-3'; NII_bot_primer (sequence of the bottom strand 1,285 to 1,251), 5'-GAG GCT GAT GGT GTT TAT GCA AAG AAA CCA CTG TG-3'; NIII_top_primer (sequence of the top strand 1,098 to 1,133), 5'-CAT ACC TCT CTC CGT ATC CTC GTA ATC ATT TTC TTG-3'; NIII_bot_primer (sequence of the bottom strand 93 to 59), 5'-TGC CTT TGT GTG CTT AAT CAC GTA TAC TCA CGT GC-3'; NIV_top_primer (sequence of the top strand 1,338 to 1,372), 5'-TTG ATA ATT AGC GTT GCC TCA TCA ATG CGA GAT CC-3'; and NIV_bot_primer (sequence of the bottom strand 297 to 260), 5'-ATT TTT ATA TGC TTT TAC AAG ACT TGA AAT TTT CCT TG-3'. The primers were radioactively labeled with [γ-^32^P] ATP by T4 polynucleotide kinase and used for primer extension reactions, as described [51,55]. The results from the indirect end-label mapping and the primer extension mapping were visualized with a Typhoon FLA 7000 biomolecular imager (GE Healthcare Life Sciences).

### Comparison of chemical cleavage sites analyzed by primer extension mapping with the published chemical map

In order to compare the primer extension signals with the published cleavage sites, we used the raw reads for the experiments, in which the cleavage reaction period was set to 20 minutes (SRR438673, SRR438674, SRR438677). Of these, the SRR438673 and SRR438674 reads were single-ended. The paired-end reads of SRR438677 were split into two groups according to their orientation, so they could be treated as single-end reads for the mapping. The reads were mapped to the reference genome R64-1-1 (www.yeastgenome.org), which is comparable to the UCSC sacCer3 assembly, using the “mem” command of BWA (http://bio-bwa.sourceforge.net, version 0.7.10-r789). The reads that were mapped to the Watson and Crick strands were selected according to the FLAG field in the resultant SAM-format files: FLAG = 0 for Watson, FLAG = 16 for Crick. The number of 5'-end nucleotides of the reads located at each genomic coordinate was calculated by piling up the reads with the “pileup” command of MACS (https://github.com/taoliu/MACS, version 2.1.1), by setting the “extsize” argument as 1. The resultant bedGraph files were converted to the wiggle format with an in-house R pipeline. The pileup counts from the three different experiments were summed at each genomic coordinate to obtain a combined score. Cleavage sites for each sequence fragment were determined by shifting the coordinates for the 5'-ends by 1 bp in the upstream direction.

Signal intensities on the gel were acquired as XY-coordinated data with the “Plot Profile” command of ImageJ (https://imagej.nih.gov/ij/). The relative position on a line drawn on the gel was in the X column, and the signal intensity was in the Y column. By visually comparing the acquired intensity with the gel images, the base coordinates of unambiguous bands were manually assigned to the corresponding X coordinates. As Taq DNA polymerase is known to add a deoxyribonucleotide at the 3'-end [60,61], we interpreted the base positions of the primer extension signals to be one base shorter than those of the control signals, which were terminated by the incorporation of a dideoxyribonucleotide. In order to obtain practical base coordinate values, the missing values in between the identified bases were filled, by assuming that they can be estimated with the linear function that passes through the two identified bases.

### Processing of published data

The redundant dyad positions determined by Brogaard et al. [42], which are available in supplementary table 3 of their report, were liftovered from the sacCer2 to sacCer3 coordinates with the liftOver utility (http://hgdownload.cse.ucsc.edu, sacCer2ToSacCer3.over.chain.gz). In total, 344,709 of the 344,720 redundant dyads were successfully liftovered. In this liftover process, dyads around the *TRP1* locus were not removed. A wiggle format file containing the genomic coordinates and the nucleosome core particle scores was generated. The MNase-seq reads (SRX059014) [62] were trimmed with the fastq_quality_trimmer command (options: -t 20 -l 36) of the FASTX toolkit (http://hannonlab.cshl.edu/fastx_toolkit/, version 0.0.14), and mapped to the sacCer3 reference genome by BWA. Bedgraph format files were generated with the pileup command of MACS (option:—extsize 100 or 147). The pair-end chemical mapping reads generated by Brogaard *et al*. (SRX127430) [42] and Henikoff *et al*. (SRX372005, SRX372006, SRX372007) [21] were mapped to the sacCer3 reference genome by BWA. Pileup counts of the 5'-end bases of the mapped reads on each strand were obtained with the genomecov command (options: -ibam -bg -5 -strand + or -) of the bedtools program suite (http://bedtools.readthedocs.io/en/latest/, version v2.26.0). Cleavage sites for each sequence fragment were determined by shifting the coordinates for the 5'-ends by 1 bp in the upstream direction. The cleavage counts for both strands were combined, smoothed as a 7-bp moving average, and stored in wiggle format files. The wiggle and bedgraph files were loaded into IGV (http://software.broadinstitute.org/software/igv/) to generate the browser views.

## Results and discussion

### Parallel mapping of nucleosomes *in vivo* with site-directed chemical cleavage and MNase digestion

**Fig 1:**
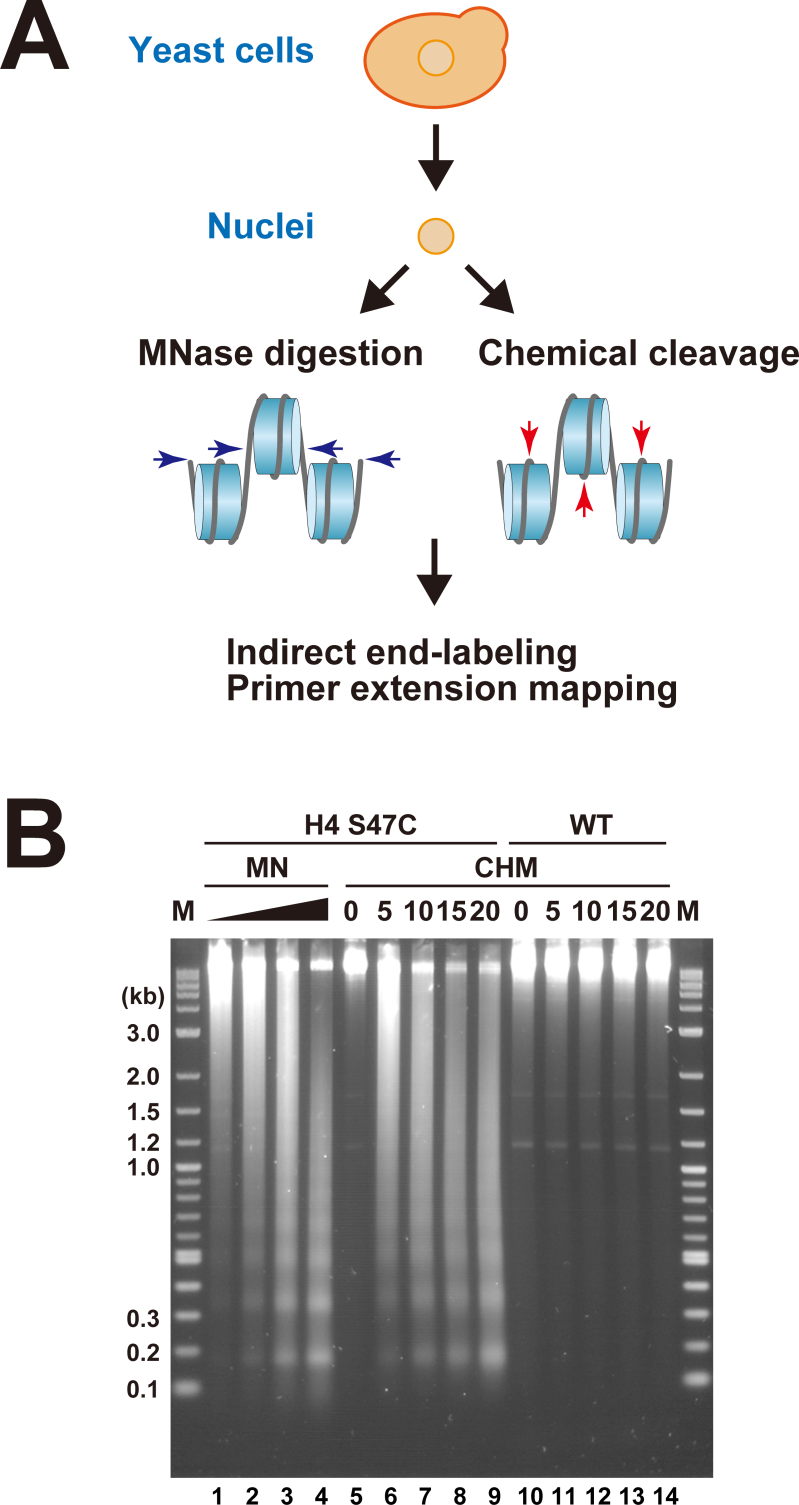
(**A) Overview of parallel mapping of nucleosomes *in vivo* in this study.** The H4 S47C strains were grown, and the nuclei were isolated. The isolated nuclei were treated with either MNase or site-directed hydroxyl radicals to digest chromatin. The DNA was purified from the digested chromatin and subjected to indirect end-labeling or primer extension mapping to detect the cleaved sites. **(B) 1.3% Agarose gel electrophoretic analysis for the genomic DNAs purified from the digested samples.** MN: DNA from the MNase-digested samples of the H4 S47C strain of MHS3002 (lanes 1–4), CHM: DNA from the site-directed chemically cleaved samples of the H4 S47C strain of MHS3002 (lanes 5–9), and the wild-type strain of Mat-alpha-YDR007W (WT, lanes 10–14). M: DNA size markers.

Since MNase has endonuclease and exonuclease activities with sequence preferences [26–30], and the sensitivity to MNase is different among nucleosomes [7–10,46,47], mapping the positions of nucleosomes by MNase digestion is often difficult, depending on their locations in the genomes. In fact, the problems of nucleosome mapping by the indirect end-labeling with MNase have been extensively discussed [29,30]. Herein, to complement the weakness of the MNase mapping methods, we applied site-directed chemical cleavages, based on the recent reports by Brogaard *et al*. [42,43], to isolated yeast nuclei (Fig 1A and Figure A in S1 File). The chemical mapping method requires the H4 S47C strain, in which the Ser47 residue in histone H4 is substituted with cysteine in *Saccharomyces cerevisiae*. The two S47C residues are located symmetrically to the DNA backbone near the SHL (SuperHelix Location = number of double helical turns from the nucleosome center) ±0.5, and are close to the dyad axis in the nucleosome [63]. For the reaction, the labeling reagent N-(1, 10-phenanthroline-5-yl) iodoacetamide was covalently linked to the S47C residue, and hydroxyl radicals were locally generated by the addition of copper and hydrogen peroxide, according to the Fenton reaction, to cleave the DNA backbone near the nucleosome center [42,43]. Thus, using the isolated nuclei as the substrate for the chemical cleavage as well as the MNase digestion, the two sides of the nucleosome can be detected by parallel mapping; *i*.*e*., the nucleosome center positions by the chemical cleavage and the linker DNA regions by the MNase digestion.

We constructed the sets of strains (MHS3001 and 3002, and MHS3005 and 3006) carrying the point mutation of S47C in the *hhf1* gene, which is one of the two genes encoding histone H4 (Table A in S1 File). The other gene *hhf2* was disrupted so that the cells only expressed the histone H4 with the S47C mutation. We first examined and determined the optimal concentrations of N-(1, 10-phenanthroline-5-yl) iodoacetamide and copper (II) chloride to add to the suspension of isolated nuclei, which were 140 μM and 10 μM, respectively. Comparing the conditions determined here with those employed with the spheroplast lysate by Brogaard *et al*. [42,43], the final concentrations of the labeling reagent and the copper (II) chloride were lowered by one-tenth and one-fifteenth, respectively. This yields a significant cost saving for the labeling reagent. In addition, the labeling reaction time was shortened to 1 hr at 4°C. This improvement of the efficiency might be due to the fact that the labeling reagent permeated readily into the isolated nuclei and that non-nuclear proteins were removed in the process of nuclei isolation. For comparison, the scheme of the parallel mapping, together with that of the chemical mapping by Brogaard *et al*. [42,43], is shown in Figure A in S1 File.

The digestions of genomic chromatin with site-directed hydroxyl radicals generated through the H4 S47C residue, as well as with MNase, are shown in Fig 1B. As the reaction time increased, more fragmentation of the yeast genomic DNA by the hydroxyl radical was detectable for the H4 S47C strain, but not for the wild-type (WT) strain, indicating that the hydroxyl radical cleavage occurred in a manner dependent on the S47C residues in histone H4. The nucleosome ladders of the hydroxyl radical-digests are comparable to those of the MNase-digests, demonstrating the integrity of the chemical reaction. These results are consistent with a recent report [64] in which hydroxyl radical cleavage was applied to macronuclei of *Tetrahymena*, as an alternative to MNase digestion. Here, we visualized the chemically and enzymatically cleaved sites by indirect end-labeling and primer extension mapping, as described below.

### Indirect end-labeling mapping of nucleosomes in TRP1ARS1

**Fig 2:**
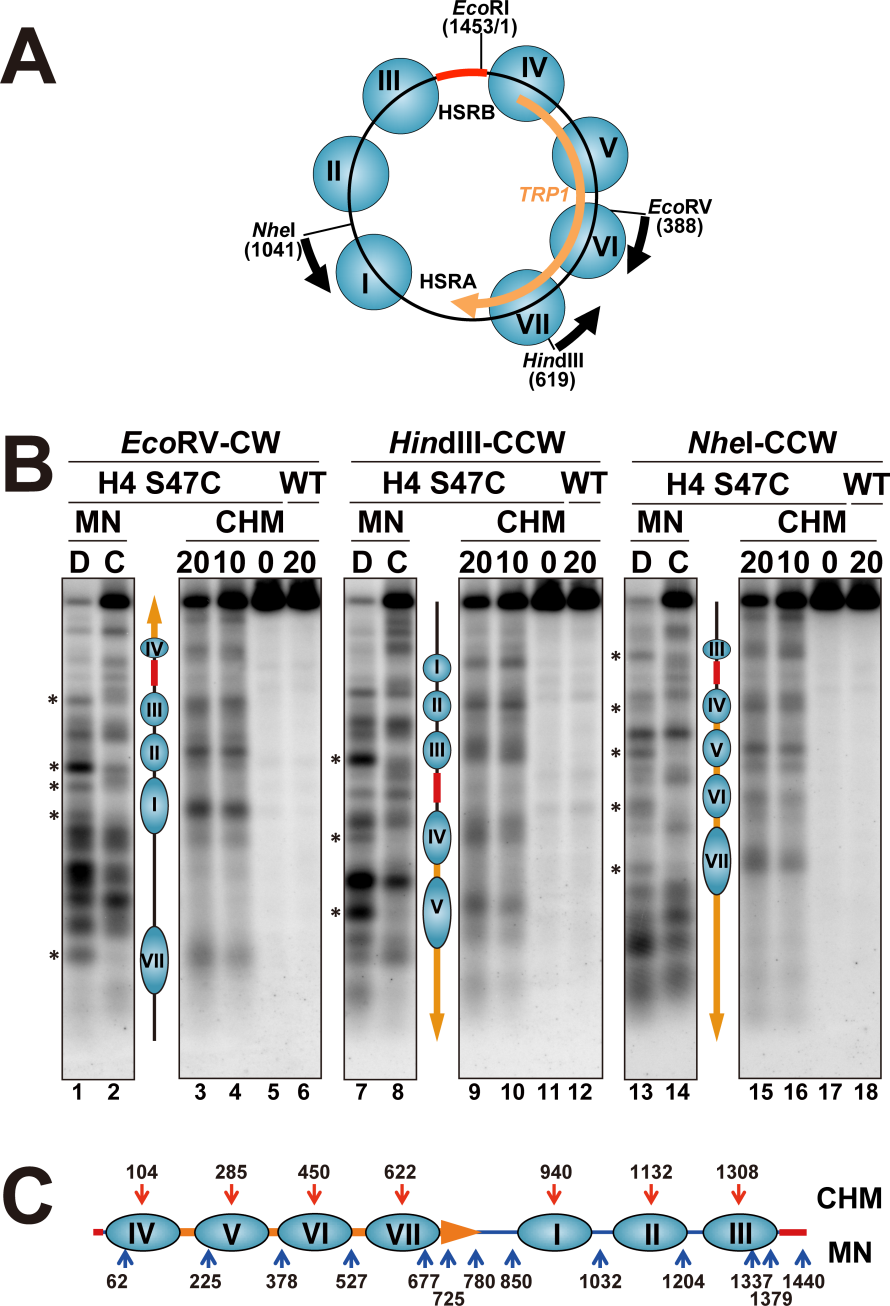
**(A) Chromatin structure of the TRP1ARS1 minichromosome.** The indirect end-labeling mapping was performed from either the *Eco*RV (388), *Hin*dIII (619) or *Nhe*I (1041) site. Position 1 is designated as the first adenosine residue in the GAATTC (*Eco*RI site) in the hypersensitive region B (HSRB). The total length of TRP1ARS1 is 1,453 bp. The ORF of the *TRP1* gene (orange arrow), HSRB (red bold bar), and the mapping direction (black arrows) are indicated. **(B) Indirect end-labeling mapping of the sites cleaved with MNase or site-directed hydroxyl radicals in the TRP1ARS1 minichromosome in the H4 S47C strain (MHS3002).** The samples were digested with *Eco*RV, *Hin*dIII or *Nhe*I and resolved by electrophoresis on a 1.2% agarose gel, and the samples were transferred to a nylon membrane for Southern blotting. The cleavage sites were detected by indirect end-labeling. Mapping from the *Eco*RV site in the clockwise direction using the *Eco*RV-*Hin*dIII probe (left, *Eco*RV-CW), from the *Hin*dIII site in the counter-clockwise direction using the *Eco*RV-*Hin*dIII probe (middle, *Hin*dIII-CCW), and from the *Nhe*I site in the counter-clockwise direction using the *Nhe*I-*Stu*I probe (right, *Nhe*I-CCW). MN, MNase-digested samples; CHM, site-directed chemically cleaved samples; D, naked DNA digested with MNase; C, chromatin in nuclei digested with MNase. The reaction times for the site-directed chemical reaction were 0, 10, and 20 minutes. WT, wild-type strain of Mat-alpha-YDR007W. The sites cleaved by MNase in naked DNA, but protected in chromatin samples are indicated by asterisks (*). The nucleosomes (blue ellipses) are shown between the MNase and chemical mapping gels in each mapping experiment, and their positions are assigned by the chemically cleaved sites. Lanes 1 and 2, 7 and 8, and 13 and 14 correspond to lanes 5 and 6 in Figures B1-B3 in S1 File, respectively. Lanes 3–6, 9–12, and 15–18 correspond to lanes 9–12 in Figures B1-B3 in S1 File, respectively. **(C) Summary of the chemically and MNase cleaved sites in the TRP1ARS1 minichromosome.** Red and blue arrows show the sites cleaved by hydroxyl radicals and MNase, respectively.

To evaluate the parallel mapping with the site-directed chemical cleavage and the MNase digestion, we chose to examine the chromatin structure of the TRP1ARS1 minichromosome, since the positions of the nucleosomes were extensively analyzed previously [65–67]. TRP1ARS1 is constructed by the circularization of the DNA fragment (1,453 bp) derived from *S*. *cerevisiae* chromosome IV, corresponding to the region from coordinates 461,740 to 463,192, which contains the *TRP1* gene with its 102-bp promoter region and the ARS1 region. Position 1 is designated as the first adenosine residue in the GAATTC (*Eco*RI site) (Fig 2A). TRP1ARS1 is a high copy plasmid maintained at 100 to 200 copies per cell [65,67]. The circularized minichromosome consists of seven nucleosomes (I to VII) and two nuclease hypersensitive regions (HSRA and HSRB), as originally mapped by Thoma *et al*. [65]. TRP1ARS1 was introduced into the H4 S47C strain of MHS3002, and the isolated nuclei were subjected to limited digestion with either MNase or the H4 S47C-directed hydroxyl radicals. The naked DNA samples were also digested with MNase as a control, while the wild-type strain (Mat-alpha-YDR007W) was used as a control for monitoring the background cleavage in the site-directed chemical reaction. The DNA was purified and digested with *Eco*RV, *Hin*dIII or *Nhe*I, and the MNase- and hydroxyl radical-cleavage sites were analyzed by indirect end-labeling, as shown in Fig 2B and Figure B in S1 File.

The positions of the nucleosomes were generally determined by comparing the MNase-cleaved sites between the chromatin and naked DNA samples. For example, in the mapping clockwise from the *Eco*RV site (lanes 1–6 in Fig 2B, and Figure B1 in S1 File), the MNase-cleaved sites in the naked DNA (indicated by asterisks) were protected in the chromatin samples, whereas the MNase-cleaved sites were detectable in the regions corresponding to the linker DNA regions. The regions protected from MNase digestion were assigned as the locations of nucleosomes I to IV (see lanes 1 and 2 in Fig 2B). These MNase-protected regions were consistently detectable in the mapping from the *Hin*dIII (lanes 7 and 8 in Fig 2B, and Figure B2 in S1 File) and *Nhe*I (lanes 13 and 14 in Fig 2B, and Figure B3 in S1 File) sites. The nuclease-hypersensitive regions HSRA and HSRB were observed between nucleosomes I and VII and between nucleosomes III and IV, respectively. We constructed a standard curve by the migrations of the DNA size markers, which were prepared by PCR using the TRP1ARS1 DNA as the template, and determined the positions of the MNase cleavage sites (the center of each band) in the TRP1ARS1 minichromosome in the H4 S47C strain (Fig 2C, blue upward arrows), which were in good agreement with the previous results [65,67]. Accordingly, the MNase digestion patterns of the wild-type and H4 S47C strains were identical to one another (compare lanes 1–4 with lanes 5–8 in Figure B in S1 File). These results confirmed that the mutation of H4 S47C has no effect on the nucleosome positioning.

Regarding the site-directed chemical mapping, the hydroxyl radicals generated through the H4 S47C residue attack the DNA backbone near the dyad axis of the nucleosome [42,43]. The positions of the nucleosomes in TRP1ARS1 were detectable in the 10 and 20 min reactions, as shown in Fig 2B (lanes 3, 4, 9, 10, 15 and 16) and Figure B in S1 File. Importantly, in the wild-type samples, which were the controls for chemical mapping, no significantly cleaved bands were observed and the pattern remained unchanged during the reaction (lanes 6, 12 and 18 in Fig 2B, also see Figure B in S1 File). The hydroxyl radical-cleaved sites were observed in the middle of the region protected by MNase for nucleosomes I, II, V, VI and VII. In contrast, the cleaved sites in nucleosomes III and IV were not located in the middle of the MNase-protected region, but were closer to the side of HSRB (*e*.*g*., see lanes 8–10 in Fig 2B). The positions of the cleaved sites (the center of each band) in each gel are summarized in Fig 2C and Table B in S1 File, together with the MNase cleavage sites.

To examine the reliability of the chemical mapping, we compared the nucleosome positions in the TRP1ARS1 minichromosome between the MNase and chemical mappings. The centers of the nucleosomes are determined as the cleaved sites by the site-directed chemical mapping (Fig 2C, red downward arrows and Table B in S1 File), while the linker regions are mapped by MNase digestion (Fig 2C, blue upward arrows). For comparison, the centers of the nucleosomes are also estimated as the midpoint of the regions protected from MNase, which are between two cleaved sites (Table B in S1 File). As seen in Table B in S1 File, the center positions of nucleosomes I and VI coincided well between the MNase and chemical mappings. For nucleosomes II, V and VII, for which the regions protected from MNase were larger than 150 bp, the distances between the center positions determined by the MNase and chemical mappings were within 20 bp. In these cases, since the regions protected from MNase were larger than the ideal nucleosomal DNA (145–147 bp), it is possible that the centers of the nucleosomes were not positioned at the midpoints of the protected regions, but instead located closer to one side. Thus, the precise positions of nucleosomes would be ambiguous for regions larger than 150 bp protected from MNase digestion. The differences in the center positions of nucleosomes III and IV between the MNase and chemical mappings were 40 bp and 37 bp, respectively, which were larger than those of the other nucleosomes. It should be noted that when nucleosomes III and IV were actually placed on the DNA, to fit the positions determined by chemical mapping, the HSRB-proximal quarters of these nucleosomal DNAs were sensitive to MNase. In fact, the width of the MNase protected region for nucleosome III was only 132 bp (Fig 2C and Table B in S1 File), which was smaller than the ideal nucleosomal DNA. This may indicate that the nucleosomes flanking the nucleosome-free region are more dynamic and/or less stable. The differences of the nucleosome positions between the MNase and chemical mapping data could also be interpreted by multiple, alternative overlapping nucleosome arrays (also called redundant positions) [29,68–70]. These points will be discussed in the High-resolution primer extension mapping section.

According to the MNase mapping results reported previously [65], the HSRB region, which was hypersensitive to MNase digestion, was assigned as a region of about 180 bp (positions 1,337 to 62 in the TRP1ARS1), and our MNase mapping results by indirect end-labeling (Fig 2) agreed well with this result. However, the chemical mapping showed that the positions of nucleosomes III and IV were 1,308 and 104 by indirect end-labeling, respectively, assigning a nucleosome-free region of only about 105 bp, given that the nucleosomes consisted of the canonical histone octamers. Thus, the use of MNase mapping alone has the potential to overestimate the size of the nucleosome-free region. Indeed, the problems and ambiguities of nucleosome mapping by indirect end-labeling with MNase have been discussed [29]. The parallel mapping with chemical and MNase cleavages is an improved method for mapping nucleosome positions by indirect end-labeling.

To check the reproducibility of the chemical mapping established here, we also performed chemical mapping of the TRP1ARS1 minichromosome using another H4 S47C strain, MHS3006, which was constructed from the FY24 strain [56]. As shown in Figure C in S1 File, the results of indirect end-labeling for the MHS3006 strain were identical to those obtained with the MHS3002 strain with the BY4742 background. Thus, the construction of the H4 S47C strains and the experimental conditions are reproducible.

### High-resolution primer extension mapping of nucleosomes in TRP1ARS1

**Fig 3:**
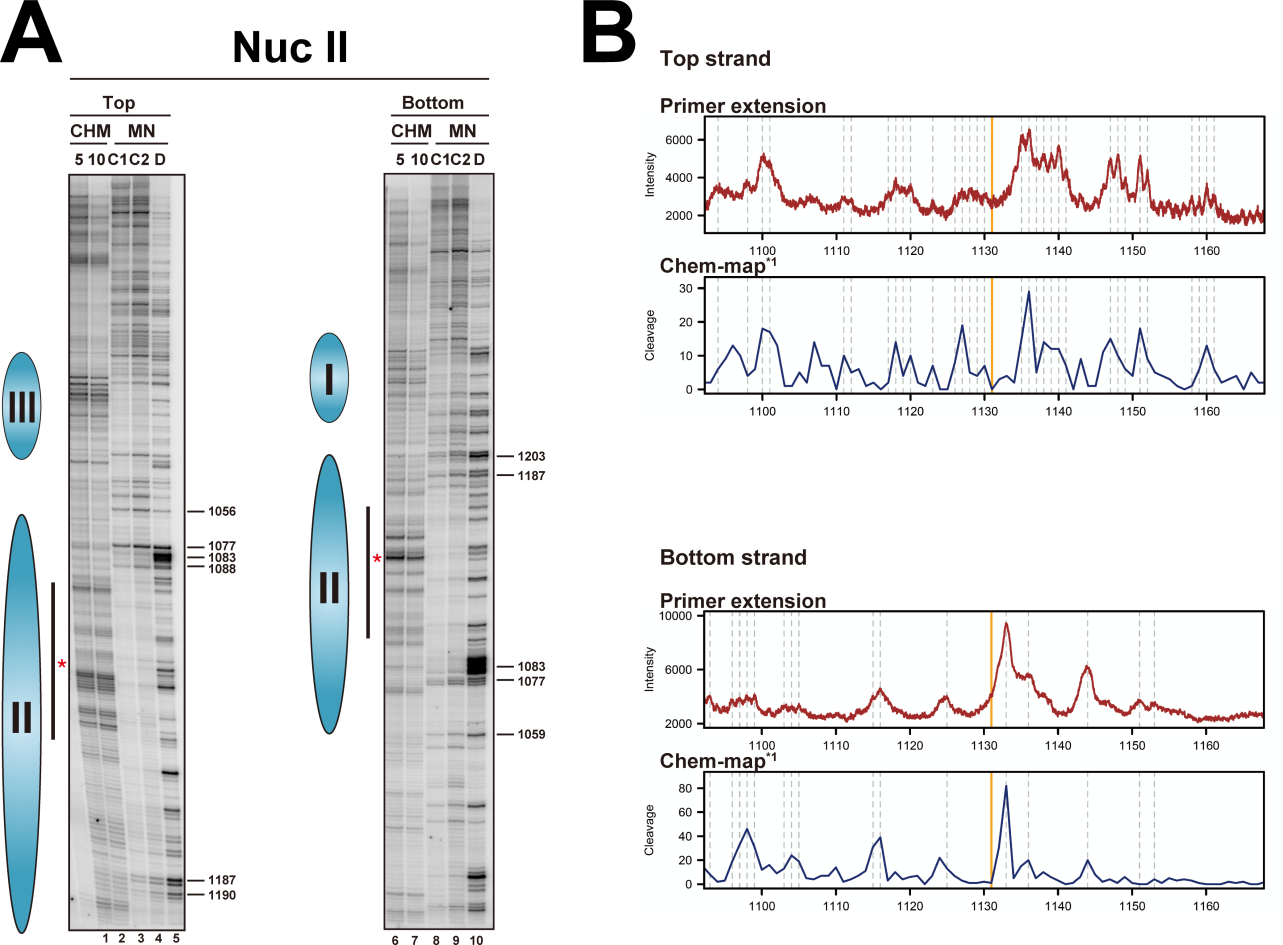
**(A) Autoradiograms of primer extension mapping of the cleaved sites in the top and bottom strands by the site-directed hydroxyl radical (CHM) and MNase (MN) in the nucleosome II region of TRP1ARS1 in the H4 S47C strain (MHS3002).** In each data set, lanes labeled 5 and 10 on the top indicate the reaction times in minutes for the hydroxyl radical cleavage, lanes labeled C1 and C2 indicate MNase digestion of isolated nuclei (chromatin) at two nuclease levels, and lanes labeled D indicate MNase digestion of the naked DNA. Lanes 1–5 correspond to lanes 9–13 in Figure D1 in S1 File; lanes 6–10 correspond to lanes 9–13 in Figure D2 in S1 File. The DNA samples are identical to those used in the indirect end-labeling mapping (Fig 2). The cleaved sites in the top and bottom strands were detected using the NucII_bot_primer and the NucII_top_primer, respectively. The locations of nucleosomes (blue ellipses) are shown on the left side of each gel, and the red asterisks indicate the center of nucleosome II, as determined by the chemically cleaved sites in the indirect end-labeling mappings (Fig 2). The numbers on the right sides of the gels indicate the characteristic cleavage sites at the edge of nucleosome II. The region analyzed densitometrically in B is shown by black bars. **(B) Comparison of the intensities of the hydroxyl radical cleaved sites with the chemical cleavage occurrence reported by Brogaard *et al*.** [42]. In each strand, the upper (Chem-map*^1^ by Brogaard *et al*. [42]) and lower (primer extension) panels show the occurrence of cleavage in the genome-wide chemical mapping (see Materials and Methods) and the densitometric analysis of the primer extension mapping in Fig 3A, respectively. The numbers of the base positions for the Chem-map*^1^ data were converted to those of TRP1ARS1 (The coordinates of 461,740 in chromosome IV correspond to position 1 in TRP1ARS1). Vertical orange lines and dashed gray lines indicate the position of the dyad determined by Brogaard *et al*. [42] and the coordinates that were visually identified on the gel, respectively.

The MNase cleavage sites in yeast minichromosomes and in genomic loci at base-pair resolution have been detected by single-cycle and multi-cycle primer extension mappings, respectively [51,53–55]. Thus, to analyze the chemical cleavage sites in more detail, we examined the chemical cleavage sites by single-cycle primer extension mapping with sequencing gel electrophoresis for the same samples used for indirect end-labeling as described above, including the samples of wild-type strains (Mat-alpha-YDR007W). The hydroxyl radicals oxidatively attack the sugar moiety, which causes the elimination of the deoxyribonucleotide, resulting in strand cleavage [35,36,38]. Thus, the cleaved sites in one strand are detectable by the primer extension of the other strand. The primer extension mapping of the chemically- and MNase-cleaved sites on each DNA strand, in the regions of nucleosome II, are shown in Fig 3A and Figures D1 and D2 in S1 File. The regions between positions 1,077 and 1,187 (the protected region was defined as 109 bp, from 1,078 to 1,187) on both the top and bottom strands (lanes 3, 4, 8 and 9 in Fig 3A) were clearly protected from MNase digestion as compared to the digestion of naked DNA, indicating the stable positioning of nucleosome II. The clearly protected region was around 109 bp, but not 145 bp, suggesting that the peripheral regions (*i*.*e*., the SHL: ±5.0~6.5) of this nucleosome were accessible to MNase. Thus, if an MNase-preferential sequence were incorporated on one side of the peripheral region, it would be cut even within a nucleosome core particle. This revealed another weakness of the MNase-dependent mapping: when the protected region is too short, since the position of the nucleosomal center cannot be determined, it is hard to determine which side of the nucleosomal DNA is more accessible to MNase.

Several chemically cleaved sites were observed around the center of the MNase-protected region in both the top and bottom strands (lanes 1, 2, 6 and 7 in Fig 3A and also see Figures D1 and D2 in S1 File). The cleavage patterns were different between the top and bottom strands, consistent with previous reports [42,43]. Again, in the samples of the wild-type strains, no significantly specific cleaved bands were observed, and the pattern remained unchanged during the time course of the reactions in primer extension mapping (WT lanes in Figure D in S1 File). The existence of the chemically cleaved sites around the center (position: 1,132) of the MNase-protected region, which spans from position 1,077 to 1,187, is consistent with the idea that both sides of the nucleosomal DNA in nucleosome II are accessible to MNase, as mentioned above.

Since the DNA sequence of TRP1ARS1 is the same as a portion (coordinates 461,740 to 463,192) of chromosome IV, we compared the band intensities of primer extension mapping with the chemical cleavage occurrence in the same sequence in the genomic locus, which was reported by Brogaard *et al*. [42]. The region was analyzed densitometrically, and is shown by the black bars in Fig 3A, where the cleaved site determined by the results of indirect end-labeling (position: 1,132) is marked with red asterisks. As shown in Fig 3B, the cleavage pattern of the primer extension mapping for each strand was in very good agreement with the previous results of the chemical cleavage occurrence in each strand (Chem-map*^1^). These results indicated that the chemical cleavage through the S47C residue of histone H4 is highly specific and reproducible, even though the experimental procedures were quite different from each other. According to previous statistical analyses of the nucleosome dyads [42,43], the dyad of nucleosome II in TRP1ARS1 was assigned to be 1,131 (orange vertical lines in Fig 3B), which coincided with the center position of 1,132 determined by indirect end labeling (Fig 2, and Table B in S1 File). Thus, although the resolution is relatively low, indirect end-labeling mapping using an agarose gel is useful for determining nucleosome positions.

**Fig 4:**
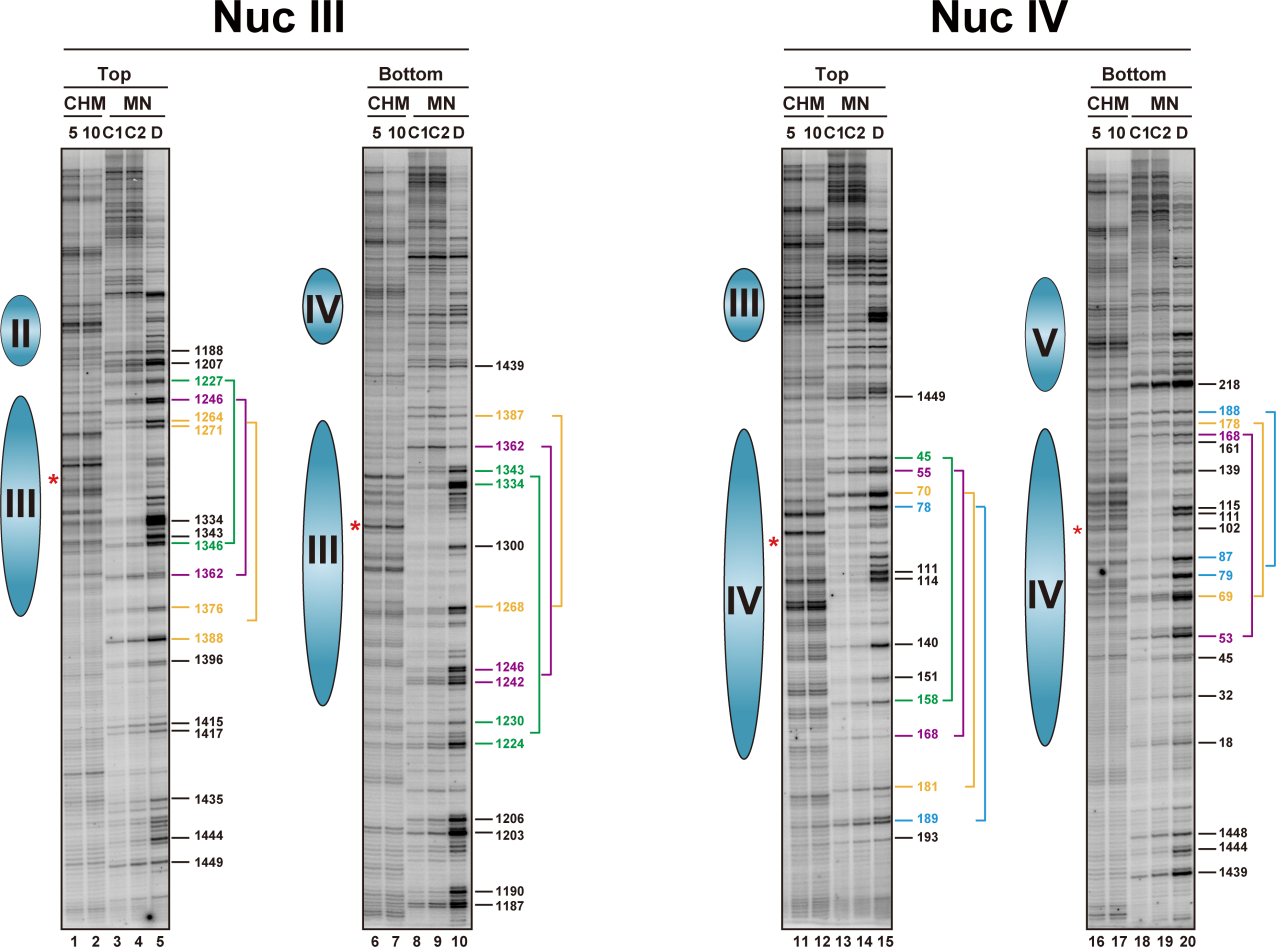
Autoradiograms of primer extension mapping of the cleaved sites in the top and bottom strands by the site-directed hydroxyl radical (CHM) and MNase (MN) in the nucleosome III (NucIII) and IV (NucIV) regions of TRP1ARS1 in the H4 S47C strain (MHS3002). In each set of data, lanes labeled 5 and 10 on top indicate the reaction times in minutes for the hydroxyl radical cleavage, lanes labeled C1 and C2 indicate MNase digestion of isolated nuclei (chromatin) at two nuclease levels, and lanes labeled D indicate MNase digestion of the naked DNA. Lanes 1–5 correspond to lanes 5–9 in Figure D3 in S1 File; lanes 6–10 correspond to lanes 9–13 in Figure D4 in S1 File; lanes 11–15 correspond to lanes 5–9 in Figure D5 in S1 File; and lanes 16–20 correspond to lanes 5–9 in Figure D6 in S1 File. The DNA samples are identical to those used in the indirect end-labeling mapping (Fig 2). The cleaved sites in the top and bottom strands of the nucleosome III regions were detected with the NucIII_bot_primer and the NucIII_top_primer, respectively, and those in the top and bottom strands of the nucleosome IV regions were detected with the NucIV_bot_primer and the NucIV_top_primer, respectively. The blue ellipses on the left side of each gel show the locations of nucleosomes, and the red asterisks indicate the center positions of the nucleosomes, as determined by indirect end-labeling of the chemically cleaved sites (Fig 2). The numbers on the right sides of the gels indicate the characteristic MNase cleavage sites in nucleosomes III and IV. Putatively assigned multi-positions of nucleosomes are shown as frames of MNase-cleaved sites on the right side of the gels: the top strand of nucleosome III, 1,227 to 1,346 (green), 1,246 to 1,362 (purple), and 1,264/1,271 to 1,376/1,388 (yellow) (lanes 3 and 4); the bottom strand of nucleosome III, 1,224/1,230 to 1,334/1,343 (green), 1,242/1,246 to 1,362 (purple), and 1,268 to 1,387 (yellow); the top strand of nucleosome IV, 45 to 158 (green), 55 to 168 (purple), 70 to 181 (yellow), and 78 to 189 (cyan); and the bottom strand of nucleosome IV, 53 to 168 (purple), 69 to 178 (yellow), and 79/87 to 188 (cyan). Each color corresponds to the same frame in the top and bottom strands.

As mentioned in the indirect end-labeling mapping section, nucleosomes III and IV showed the characteristic feature that the HSRB-proximal quarters of these nucleosomal DNAs were sensitive to MNase, when the nucleosomal centers were assigned by the chemically cleaved sites. Thus, we also analyzed nucleosomes III and IV by primer extension mapping, as shown in Fig 4 and Figures D3-D6 in S1 File. For nucleosome III, the regions clearly protected from MNase digestion were 74 bp (between the band positions 1,271 and 1,346) and 65 bp (positions 1,268 to 1,334) in the top and bottom strands, respectively (lanes 3, 4, 8 and 9 in Fig 4), which were narrow as compared to those in nucleosome II. In contrast, the region containing chemically cleaved sites was about 75 bp (positions 1,271 to 1,346 and 1,268 to 1,343 in the top and bottom strands, respectively, lanes 1, 2, 6 and 7 in Fig 4), which was wide as compared to that of nucleosome II. Similarly, the MNase-protected regions of nucleosome IV were 87 bp (between the band positions 70 and 158) and 80 bp (between the band positions 87 to 168) in the top and bottom strands, respectively (lanes 13, 14, 18 and 19 in Fig 4). The region containing chemical cleavage sites for nucleosome IV was also wide (positions 70–140). Thus, the patterns of the MNase and chemical mapping for nucleosomes III and IV were different from those for nucleosome II.

One possible explanation for this difference is that nucleosomes III and IV form at multiple, alternative overlapping positions, since there is invadable space in the nucleosome-free region of HRSB. It is possible that the positions of nucleosomes III and IV are perturbed by transcription, since the region from 1 to 102 is the promoter region of the *TRP1* gene, which is constitutively expressed [71]. Based on the results obtained with nucleosome II, for which the MNase protected region was about 110 bp, it can be interpreted that several frames of protected regions exist for nucleosome III, which were putatively assigned as the regions of 1,227 to 1,346 (green bracket), 1,246 to 1,362 (purple bracket), and 1,264/1,271 to 1,376/1,388 (yellow bracket) for the top strand (lanes 3 and 4 in Fig 4), and 1,224/1,230 to 1,334/1,343 (green bracket), 1,242/1,246 to 1,362 (purple bracket), and 1,268 to 1,387 (yellow bracket) for the bottom strand (lanes 8 and 9 in Fig 4). Similarly, in nucleosome IV, the multiple frames are considered to be 45 to 158 (green bracket), 55 to 168 (purple bracket), 70 to 181 (yellow bracket), and 78 to 189 (cyan bracket) for the top strand (lanes 13 and 14 in Fig 4), and 53 to 168 (purple bracket), 69 to 178 (yellow bracket), and 79/87 to 188 (cyan bracket) for the bottom strand (lanes 18 and 19 in Fig 4). Thus, it is possible that such redundant positioning of nucleosomes causes the chemically cleaved sites to spread out over a wide area in the MNase-protected region. Another possibility is that the nucleosomes flanked by the nucleosome-free region do not adopt canonical structures, as subnucleosomes have been proposed to flank the nucleosome-free regions [11]. In addition, an altered nucleosome structure may be formed dynamically in the promoter of a transcribed gene.

### Comparison of nucleosome positions in the TRP1ARS1 minichromosome with those in the genomic *TRP1* locus

**Fig 5:**
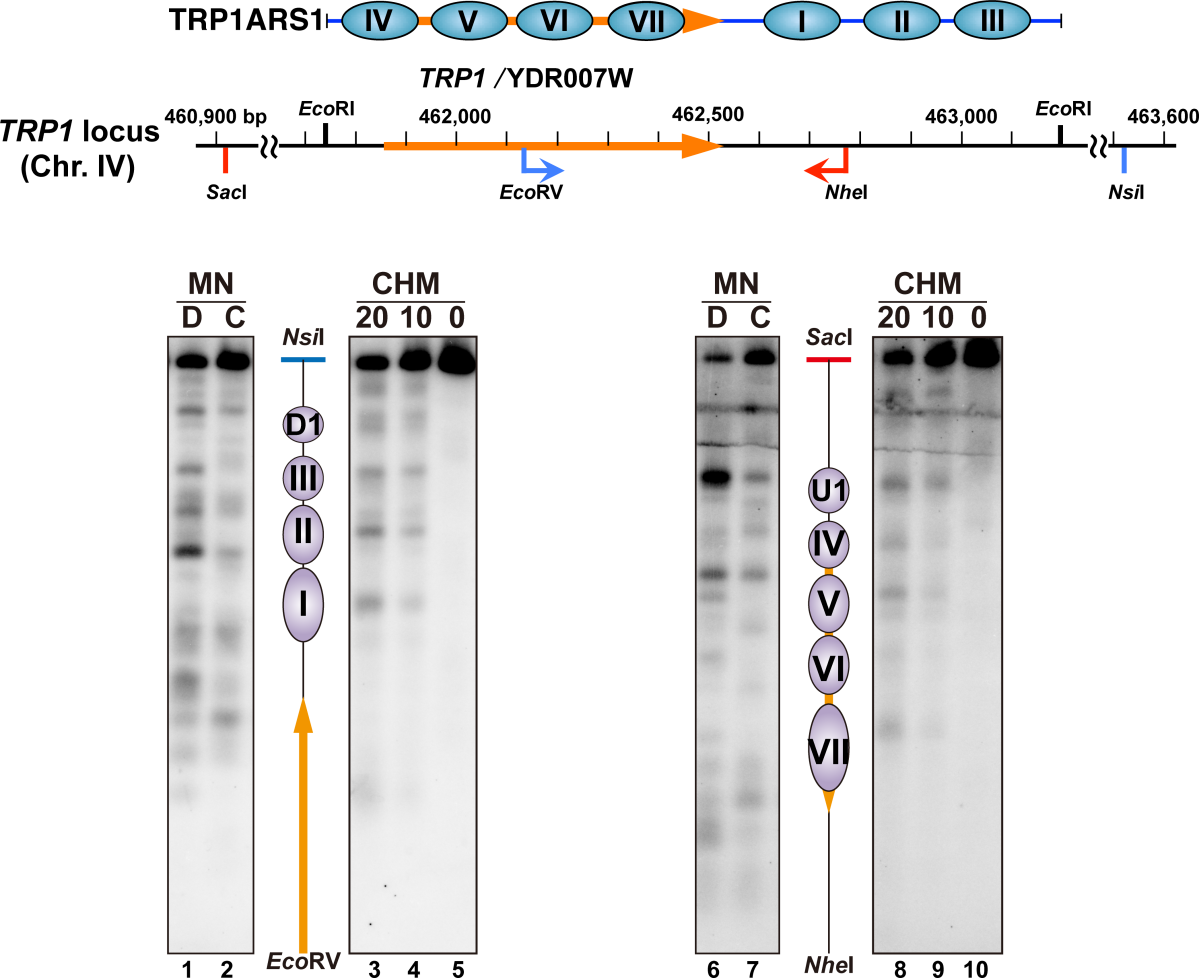
Indirect end-labeling mapping of the sites cleaved with MNase (MN) or site-directed hydroxyl radicals (CHM) in the genomic *TRP1* locus in the H4 S47C strain (MYA-4902). The locations of the *TRP1* locus in chromosome IV and in the TRP1ARS1 minichromosome are shown in the upper portion. The samples double-digested with *Eco*RV and *Nsi*I or *Nhe*I and *Sac*I were resolved by electrophoresis on a 1.2% agarose gel, and the samples were transferred to a nylon membrane for Southern blotting. The cleaved sites were mapped from the *Eco*RV site (left) and from the *Nhe*I site (right) using the *Eco*RV-*Hin*dIII and *Nhe*I-*Stu*I probes, respectively. In each set of data, lanes labeled 10 and 20 indicate the reaction times in minutes for the hydroxyl radical cleavage, lanes labeled C indicate MNase digestion of isolated nuclei (chromatin), and lanes labeled D indicate MNase digestion of the naked DNA. Purple ellipses labeled I to VII correspond to nucleosomes I to VII in the TRP1ARS1 minichromosome, and purple ellipses labeled U1 and D1 indicate the nucleosomes upstream and downstream of the TRP1ARS1 sequence region. Lanes 1 to 5 correspond to lanes 4, 5, 7, 8, 9 in Figure E1 in S1 File, respectively. Lanes 6–10 correspond to lanes 4, 5, 7, 8, 9 in Figure E2 in S1 File, respectively.

**Fig 6:**
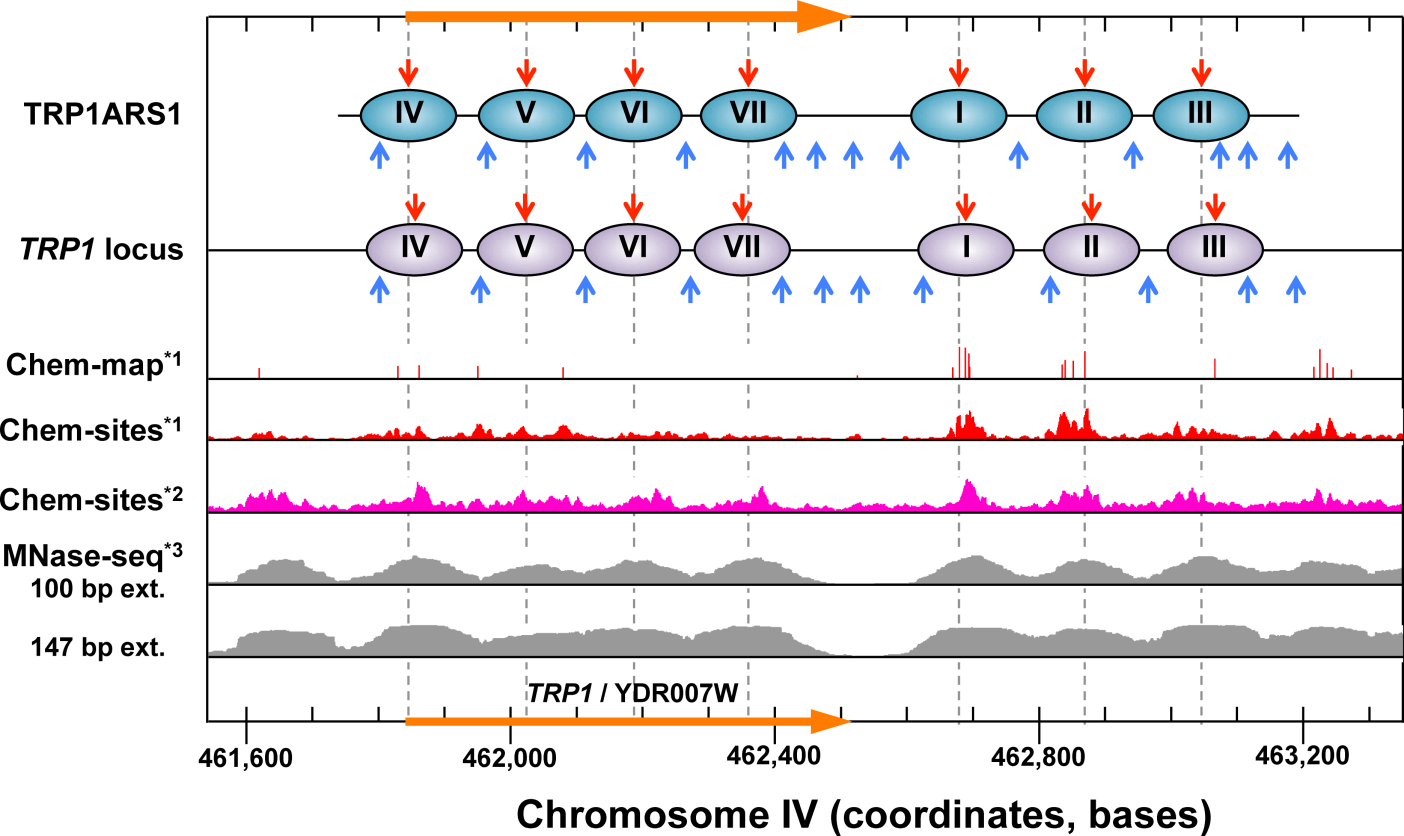
Locations of nucleosomes in TRP1ARS1 and in the *TRP1* locus determined in this work, in comparison to those in the *TRP1* locus detected by Chem-map and MNase-seq analyses. Nucleosome positions in TRP1ARS1 and in the *TRP*1 locus are summarized in Table B in S1 File. Chem-map*^1^: Dyad positions determined by Brogaard *et al*. [42] are shown by red vertical bars. The heights of the bars indicate the nucleosome core particle scores for each dyad. Pileup views of the chemical cleavages on both the Watson and Crick strands are shown: Brogaard *et al*. (Chem-sites*^1^) [42] and Henikoff *et al*. (Chem-sites*^2^) [21]. MNase-seq*^2^: Nucleosome positions analyzed by Tsankov *et al*. [62] are shown as a pileup view of reads, in which the reads are extended to a length of 100 bp to visually emphasize the center of nucleosomes, and to a length of 147 to show the nucleosome occupancy.

To compare the nucleosome positions in the circular TRP1ARS1 minichromosome with those in the genome, we analyzed the chromatin structure around the genomic *TRP1* locus in the H4 S47C strain (MYA-4902), which carries the wild-type *TRP1* gene. The DNA sequence of TRP1ARS1 corresponds to the *TRP1* locus between two *Eco*RI sites, which is between 461,740 and 463,192 on chromosome IV. This region was analyzed by indirect end-labeling from two directions: one was from the *Eco*RV site to the *Nsi*I site, and the other was from the *Nhe*I site to the *Sac*I site, as shown in Fig 5 and Figure E in S1 File. Chemical and MNase mappings revealed the presence of positioned nucleosomes corresponding to nucleosomes I to VII in TRP1ARS1, and the center positions of these nucleosomes in the genomic locus were in good agreement with those in TRP1ARS1 (Fig 6 and Table B in S1 File). These results support the idea that the DNA sequence is the major determinant for the positioning of these nucleosomes [66].

We further compared our results with the previous genome-wide chemical mapping [21,42] and MNase-Seq [62] results, as shown in Fig 6. The nucleosome positions determined by indirect end-labeling of the MNase and chemical cleavage sites in the TRP1ARS1 minichromosome and the genomic *TRP1* locus were consistent with those identified by the MNase-seq analysis. There was also good agreement for the dyad positions of nucleosomes I to III between indirect end-labeling and the genome-wide chemical map reported by Brogaard *et al*. [42] (Chem-map*^1^). In contrast, the dyad position of nucleosomes IV to VII were not clearly detected by the Chem-map*^1^, while they were detectable by indirect end-labeling in the genomic *TRP1* locus. Since the *TRP1* gene is transcribed constitutively in the absence and presence of the amino acid tryptophan [71], the structures and positions of nucleosomes IV to VII would be altered dynamically in the coding region. Consistent with this idea, the positions of nucleosomes V to VII became more ambiguous in the MNase-seq analysis, when the reads were extended from a length of 100 bp to 147 bp. Thus, there is a possibility that the nucleosomal DNA being transcribed is partially unwrapped and thus beyond the reach of the hydroxyl radical generated around the S47C residue of H4 in the *TRP1* gene (nucleosomes IV to VII). As a result, the mono-nucleosome-size DNA fractions analyzed by Brogaard *et al*. [42] would not include all of the cleavage fragments derived from the juxtaposed nucleosomes in this region, and hence it is considered that these nucleosomes tend to be missing in the Chem-map*^1^ reported by Brogaard *et al*. [42].

To explore this possibility, we also examined the genome-wide chemical map data reported by Henikoff *et al*. [21] (Chem-sites*^2^ in Fig 6). Interestingly, as compared to the results by Brogaard *et al*. [42] (Chem-sites*^1^ in Fig 6), the chemically cleaved sites in the region of nucleosomes IV to VII were more pronounced in the results by Henikoff *et al*. [21]. To understand this difference in the chemical cleavage site profiles, we examined the sizes of the DNA fragments used in their analyses. As shown in Figure F in S1 File, 98% of the DNA fragments used in Brogaard *et al*. were in the range from 100 to 200 bp in length, whereas the fragments analyzed by Henikoff *et al*. showed a wider distribution, and probably contained fragments from juxtaposed nucleosomes with longer linkers. The differences in the chemical cleavage site profiles between Brogaard *et al*. and Henikoff *et al*. may be explained by the differences in the distributions of the DNA fragment sizes used in their studies. Taken together, the size of the DNA fragments used in the NGS analysis affects the profile of the chemical cleavage sites.

It should be noted that indirect end-labeling using partially digested samples without size-selection, can detect the H4 site-directed chemical cleavages in nucleosomes IV to VII in the transcribed *TRP1* genes (Fig 5 and Figure E in S1 File). Consistent with the above interpretation, the chemical cleavage signals of nucleosomes IV to VII (especially IV and VI) in the indirect end-labeling result appeared to be relatively low as compared to that of the upstream nucleosome U1 (lanes 8 and 9 in Fig 5), possibly due to the transcription of *TRP1*.

In summary, we established a gel-based parallel mapping method to analyze the positions and dynamic natures of nucleosomes *in vivo*, using isolated nuclei as the substrates for site-directed chemical cleavage and for MNase digestion. The nucleosomes were mapped more accurately by this parallel mapping method, as compared to the MNase mapping alone. With this method, as the center of a given nucleosome is determined by chemical cleavage, researchers can study the dynamic nature of the wrapped DNA in terms of the MNase accessibility. Using this analysis, we showed that the structures and dynamics of individual nucleosomes strongly depend on their locations and circumstances in the TRP1ARS1 minichromosome as well as in the *TRP1* locus. The method described here does not require the NGS technologies, but uses common gel electrophoresis equipment. Indeed, mapping data in a whole genome is not always necessary; instead, the analysis of a specific locus under several conditions and/or in many mutant cells is often needed. Parallel mapping by indirect end-labeling will be useful for these analyses.

## Supporting information

S1 File Supplementary Tables and Figures. (PDF)
