# Supplementary material for: Parallel mapping with site-directed hydroxyl radicals and micrococcal nuclease reveals structural features of positioned nucleosomes in vivo
Source: PLoS One. 2017 Oct 26;12(10):e0186974. doi: 10.1371/journal.pone.0186974 (PMC5658119; doi:10.1371/journal.pone.0186974)
Supplement: S1 File — (PDF) [file pone.0186974.s001.pdf]

Supporting information

Parallel mapping with site-directed hydroxyl radicals and micrococcal nuclease reveals structural features of positioned nucleosomes *in vivo*

Tomohiro Fuse, Koji Katsumata, Koya Morohoshi, Yukio Mukai, Yuichi Ichikawa,
Hitoshi Kurumizaka, Akio Yanagida, Takeshi Urano, Hiroaki Kato and Mitsuhiro Shimizu

S1 File. Supplementary Tables and Figures.

Table A. Yeast strains used in this study.

Table B. Summary of the cleaved sites analyzed by the parallel mapping with indirect end-labeling.

Figure A. The scheme of the parallel mapping established in this study (Upper panel), together with that of the chemical mapping by Brogaard *et al*. (*Nature*, 486, 496-501, 2012; *Meth. Enzymol*., 513, 315-334, 2012) (Lower panel).
The differences in the experimental procedures and conditions between our analyses and those of Brogaard *et al*. are shown in red. OP, N-(1,10 phenanthroline-5-yl) iodoacetamide; PMSF, phenylmethanesulfonyl fluoride; fc, final concentration.

Figure B. Whole data for the indirect end-labeling mapping of MNase (MN) and site-directed chemical cleavage sites (CHM) in TRP1ARS1 in the H4 S47C strains of MHS3002, from the *Eco*RV site (position 388) in the clockwise direction using the *Eco*RV-*Hin*dIII probe (B1), from the *Hin*dIII site (position 619) in the counter-clockwise direction using the *Eco*RV-*Hin*dIII probe (B2), and from the *Nhe*I site (position 1041) in the counter-clockwise direction using the *Nhe*I-*Stu*I probe (B3).
Position 1 is designated as the first adenosine residue in the GAATTC (*Eco*RI site) in the HSRB, and the total length of TRP1ARS1 is 1,453 bp. The ORF of the *TRP1* gene (orange arrow), HSRB (red bold bar), and the mapping direction (black arrows) are indicated. Lanes labeled “C1, C2 and C3” indicate MNase digestions of isolated nuclei at three nuclease concentrations, and lanes labeled “D” indicate digestion of the naked DNA as a control. Lanes labeled “0, 10 and 20” indicate the reaction times for the site-directed chemical reaction in minutes. The sizes of markers are shown on the left side of the gel in bp (position numbers). WT, wild-type strain (Mat-alpha-YDR007W); H4 S47C, H4 S47C strain (MHS3002). The sites cleaved by MNase in naked DNA, but protected in chromatin samples, are indicated by asterisks (*). Lanes 5 and 6 in Figures B1-B3 correspond to lanes 1, 2, 7, 8, 13 and 14 in Fig. 2B. Lanes 9-12 in Figures B1-B3 correspond to lanes 3-6, 9-12 and 15-18 in Fig. 2B.

Figure C. Whole data for the indirect end-labeling mapping of MNase (MN) and site-directed chemical cleavage sites (CHM) in TRP1ARS1 in the H4 S47C strains of MHS3002 and 3006, which have the genetic backgrounds of BY4742 and FY24, respectively.
The samples were digested with *Eco*RV, *Hin*dIII or *Nhe*I and resolved by electrophoresis on a 1.2% agarose gel, and the samples were transferred to a nylon membrane for Southern blotting. The cleavage sites were detected by indirect end-labeling. The mapping direction is from the *Eco*RV site (position 388) in the clockwise direction, using the *Eco*RV-*Hin*dIII probe. Lanes labeled “C1 and C2” indicate MNase digestions of isolated nuclei at two nuclease concentrations, and lanes labeled “D” indicate digestion of the naked DNA as a control. Lanes labeled “0, 5, 10, 15 and 20” indicate the reaction times for the site-directed chemical reaction, in minutes. The sizes of markers are shown on the left side of the gel in bp (position numbers). WT, wild-type strain (FY24); H4 S47C, H4 S47C strains (MHS3002 and 3006).

Figure D. Whole data for the primer extension mapping of MNase (MN) and site-directed chemical cleavage sites (CHM) in the TRP1ARS1 minichromosome.
Sequencing ladders for T, G, C and A, with reactions terminated by the complementary dideoxynucleotides ddA, ddC, ddG and ddT, respectively. Lanes labeled “C1 and C2” indicate MNase digestions of isolated nuclei at two nuclease concentrations, and lanes labeled “D” indicate digestion of the naked DNA as a control. Lanes labeled “0, 5 and 10” indicate the reaction times for the site-directed chemical reaction in minutes. WT, (Mat-alpha-YDR007W); H4 S47C, H4 S47C strain (MHS3002).
(D1) and (D2) The cleaved sites on the top and bottom strands of the nucleosome II region were detected using the NucII_bot_primer and the NucII_top_primer, respectively. Lanes 9-13 in Figures D1 and D2 correspond to lanes 1-5 and lanes 6-10 in Fig. 3A, respectively.
(D3) and (D4) The cleaved sites on the top and bottom strands of the nucleosome III region were detected using the NucIII_bot_primer and the NucIII_top_primer, respectively. Lanes 5-9 in Figure D3 and lanes 9-13 in Figure D4 correspond to lanes 1-5 and lanes 6-10 in Fig. 4, respectively.
(D5) and (D6) The cleaved sites on the top and bottom strands of the nucleosome IV region were detected using the NucIV_bot_primer and the NucIV_top_primer, respectively. Lanes 5-9 in Figures D5 and D6 correspond to lanes 11-15 and lanes 16-20 in Fig. 4, respectively.

Figure E. Whole data for the indirect end-labeling mapping of MNase (MN) and site-directed chemical cleavage sites (CHM) from either the *Eco*RV (A) or *Nhe*I site (B) in the *TRP1* locus in chromosome IV in the H4 S47C strains (MYA-4902).
The samples were digested with *Eco*RV or *Nhe*I and resolved by electrophoresis on a 1.2% agarose gel, and the samples were transferred to a nylon membrane for Southern blotting. The cleaved sites were mapped from the *Eco*RV site (E1), and from the *Nhe*I site (E2), using the *Eco*RV-*Hin*dIII and *Nhe*I-*Stu*I probes, respectively. Lanes labeled “C1 and C2” indicate MNase digestions of isolated nuclei at two nuclease concentrations, and lanes labeled “D” indicate digestion of the naked DNA as a control. Lanes labeled “0, 10 and 20” indicate the reaction times for the site-directed chemical reaction in minutes. The sizes of markers are shown on the left side of the gel in bp (coordinates numbers). Lanes 4, 5 and 7-9 in Figures E1 and E2 correspond to lanes 1-5 and 6-10 in Fig. 5, respectively.

Figure F. Length distributions of fragments studied in previous genome-wide chemical cleavage studies.
Distributions of fragment lengths studied by Brogaard *et al*. [42] and Henikoff *et al*. [21] are shown. Among the fragments studied by Brogaard *et al*., 98% fell in a narrow window, ranging from 100 to 200 bp, as the mono-nucleosome-size bands were subjected to sequencing. In contrast, the fragments studied by Henikoff *et al*. showed a wider distribution, and probably contained fragments from juxtaposed nucleosomes with longer linkers.

Table A. Yeast stains used in this study

Strains	Genotypes	Sourse
BY4741	*MAT* ***a*** *his3 Δ1 leu2 Δ0 met15 Δ0 ura3 Δ0*	Open Biosystems
BY4742	*MAT α his3 Δ1 leu2 Δ0 lys2 Δ0 ura3 Δ0*	Open Biosystems
Mat-alpha-YBR009C	*MAT α his3 Δ1 leu2 Δ0 lys2 Δ0 ura3 Δ0 hhf1::KanMX*	Open Biosystems
Mat-alpha-YDR007W	*MAT α his3 Δ1 leu2 Δ0 lys2 Δ0 ura3 Δ0 trp1::KanMX*	Open Biosystems
MYA-4902	*MAT* ***a*** *his3 Δ1 leu2 Δ0 met15 Δ0 ura3 Δ0 hhf1::S47C hhf2::URA3*	ATCC
MHS3001	*MAT* ***a*** *his3 Δ1 leu2 Δ0 met15 Δ0 ura3 Δ0 trp1::KanMX hhf1::S47C hhf2::URA*[illegible]	this study
MHS3002	*MAT α his3 Δ1 leu2 Δ0 lys2 Δ0 ura3 Δ0 trp1::KanMX hhf1::S47C hhf2::URA3*	this study
FY23	*MAT* ***a*** *ura3-52 trp1 Δ63 leu2 Δ1*	CSHL[*1]
FY24	*MAT α ura3-52 trp1 Δ63 leu2 Δ1*	CSHL[*1]
MHS3003	*MAT* ***a*** *ura3-52 trp1 Δ63 leu2 Δ1 hhf2::KanMX*	this study
MHS3004	*MAT α ura3-52 trp1 Δ63 leu2 Δ1 hhf1::S47C*	this study
MHS3005	*MAT* ***a*** *ura3-52 trp1 Δ63 leu2 Δ1 hhf1::S47C hhf2::KanMX*	this study
MHS3006	*MAT α ura3-52 trp1 Δ63 leu2 Δ1 hhf1::S47C hhf2::KanMX*	this study

[*1] Strains were obtained from the Yeast Genetics course at Cold Spring Harbor Laboratory.

Table B. Summary of the cleaved sites analyzed by the parallel mapping with indirect end-labeling.

Mapping method	Chemical (CHM)		MNase (MN)			
samples	TRP1ARS1 minichromosome	Genomic *TRP1* locus	TRP1ARS1 minichromosome		Genomic *TRP1* locus	
	Cleaved sites as nucleosome center*1	Cleaved sites as nucleosome center*2	Cleaved sites as linker DNA*1	Midpoint between the cleaved sites	Cleaved sites as linker DNA*2	Midpoint between the cleaved sites
			62<br>(461,801)		62<br>(461,801)	
Nuc IV	104<br>(461,843)	117<br>(461,856)		144<br>(461,883)		139<br>(461,878)
			225<br>(461,964)		216<br>(461,955)	
Nuc V	285<br>(462,024)	284<br>(462,023)		302<br>(462,041)		296<br>(462,035)
			378<br>(462,117)		376<br>(462,115)	
Nuc VI	450<br>(462,189)	449<br>(462,188)		453<br>(462,192)		455<br>(462,194)
			527<br>(462,266)		534<br>(462,273)	
Nuc VII	622<br>(462,361)	613<br>(462,352)		602<br>(462,341)		604<br>(462,343)
HSRA			677<br>(462,416)		673<br>(462,412)	
			725<br>(462,464)		736<br>(462,475)	
			780<br>(462,519)		790<br>(462,529)	
			850<br>(462,589)		885<br>(462,624)	
Nuc I	940<br>(462,679)	950<br>(462,689)		941<br>(462,680)		982<br>(462,721)
			1,032<br>(462,771)		1,079<br>(462,818)	
Nuc II	1,132<br>(462,871)	1,143<br>(462,882)		1,118<br>(462,857)		1,154<br>(462,893)
			1,204<br>(462,943)		1,228<br>(462,967)	
Nuc III	1,308<br>(463,047)	1,329<br>(463,068)		1,271<br>(463,010)		1,304<br>(463,043)
HSRB			1,337<br>(463,076)			
			1,379<br>(463,118)		1,379<br>(463,118)	
			1,440<br>(463,179)		1,453<br>(463,192)	

*1 The position numbers of bp in the DNA sequence of TRP1ARS1 are shown together with their corresponding coordinates in chromosome IV, in parentheses. The cleaved sites were determined from the results of Fig. 2: For Nuc II, III, IV and V, determined as averages of measurements in the mappings from the *Eco* RV, *Hin* dIII and *Nhe* I sites, for Nuc I, determined as averages of measurements in the mappings from the *Eco* RV and *Hin* dIII sites, for Nuc VI and VII, determined by the mapping from the *Nhe* I site. The variations in measurements are the averages ± 20 bp.

*2 The position numbers of base-pairs in the DNA sequence of TRP1ARS1 are shown together with their corresponding coordinates in chromosome IV, in parentheses. The cleaved sites were determined from the results of Fig. 5: for Nuc I, II and III, determined in the mapping from the *Eco* RV site, for Nuc IV to VII, determined in the mapping from the *Nhe* I site.

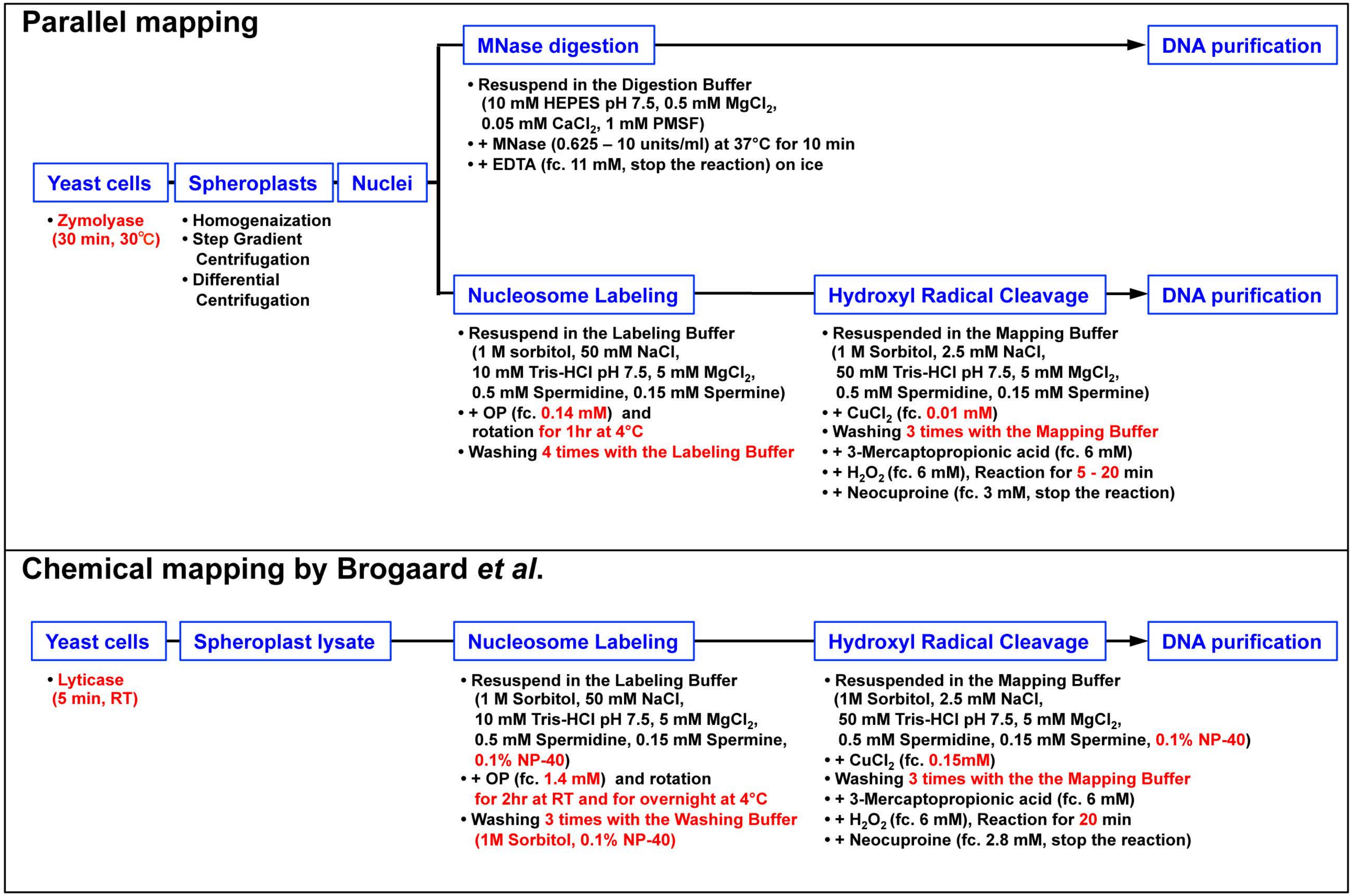

Fuse et al., Figure A

B1

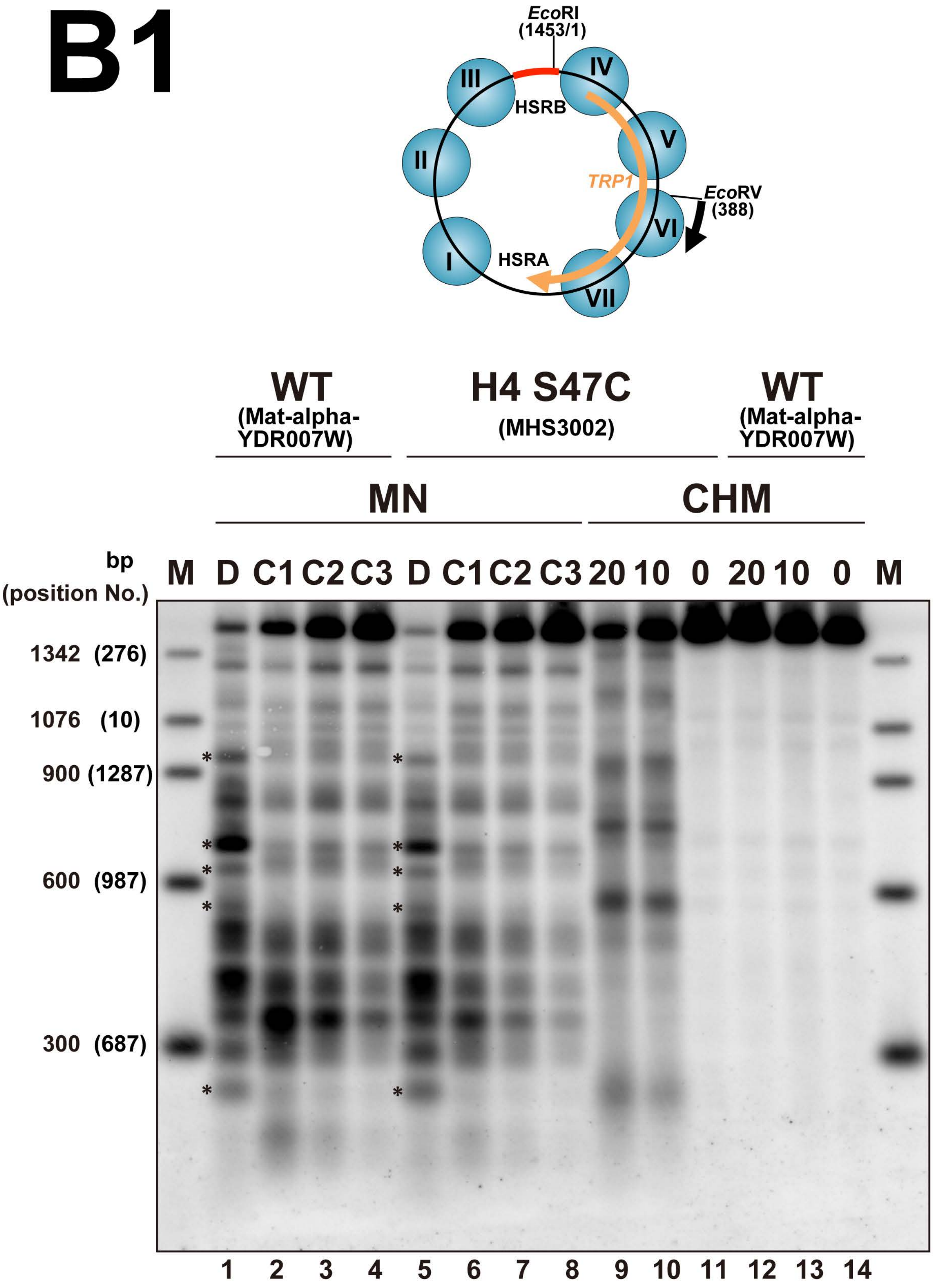

Fuse et al., Figure B1

B2

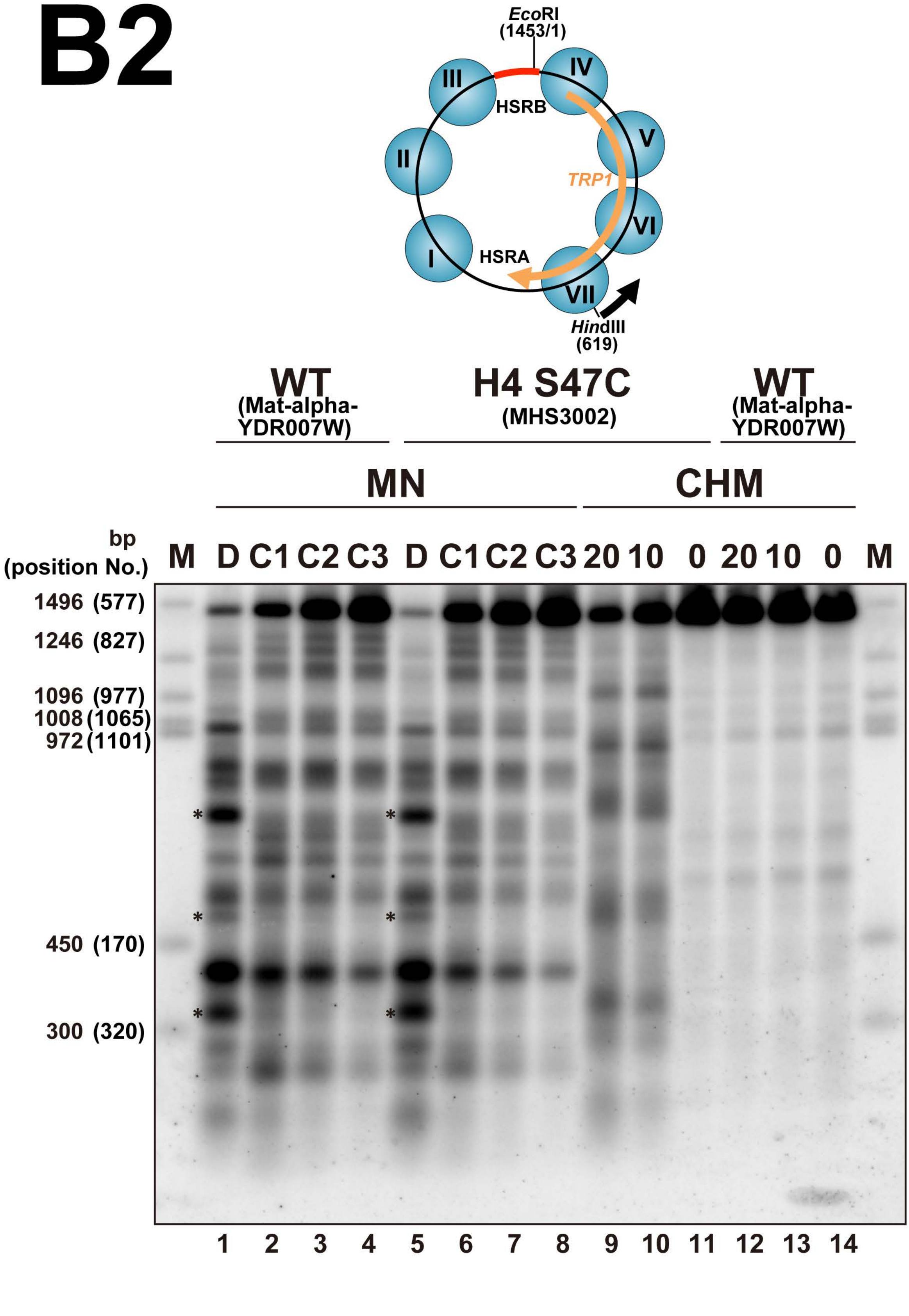

Fuse et al., Figure B2

B3

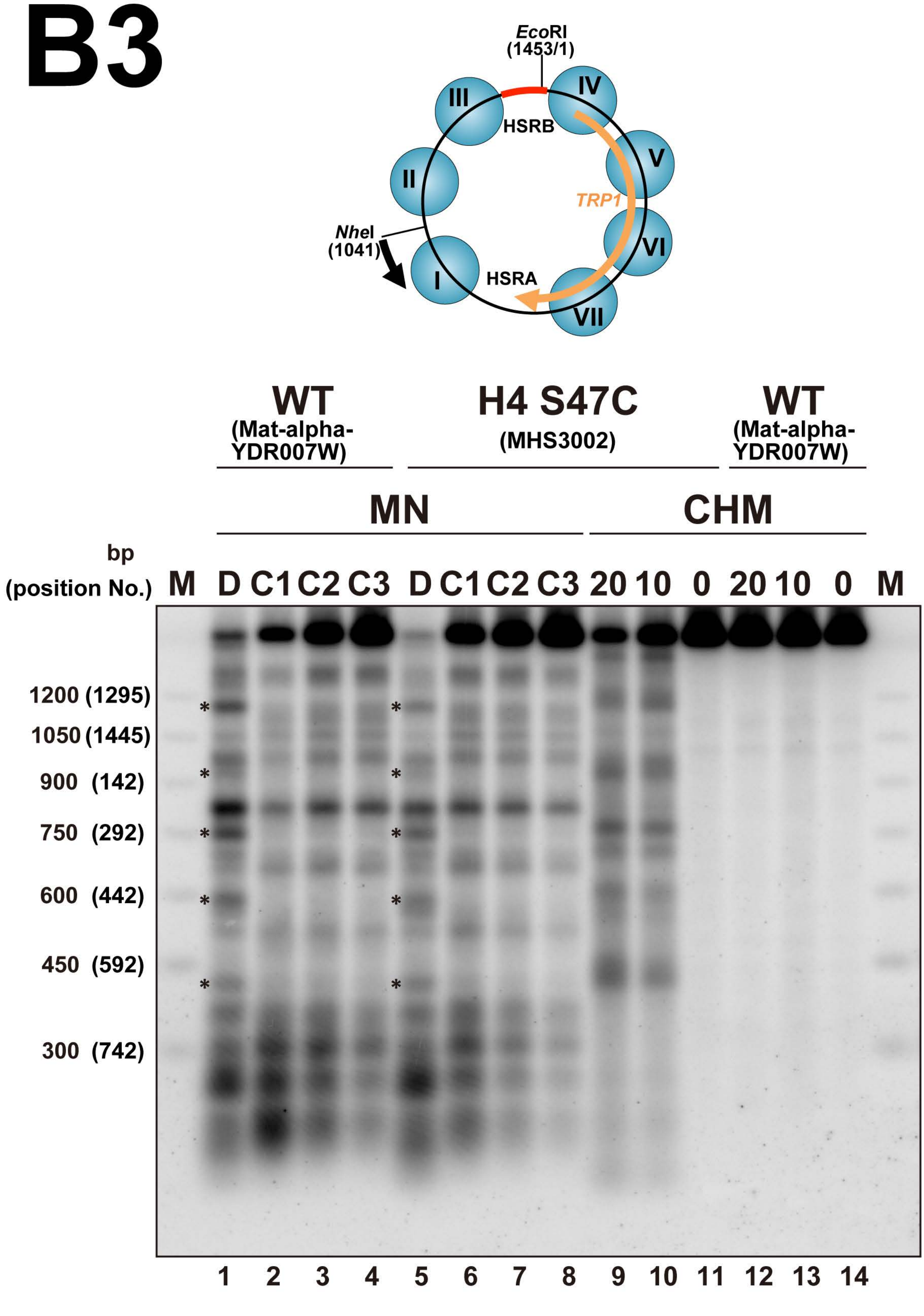

Fuse et al., Figure B3

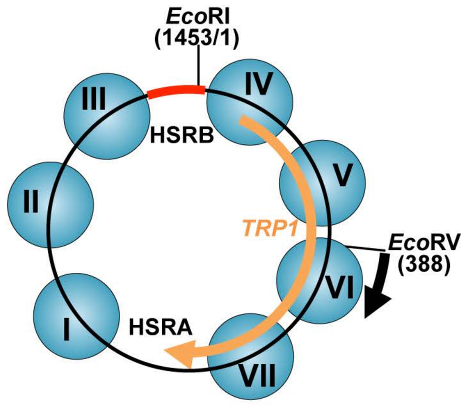

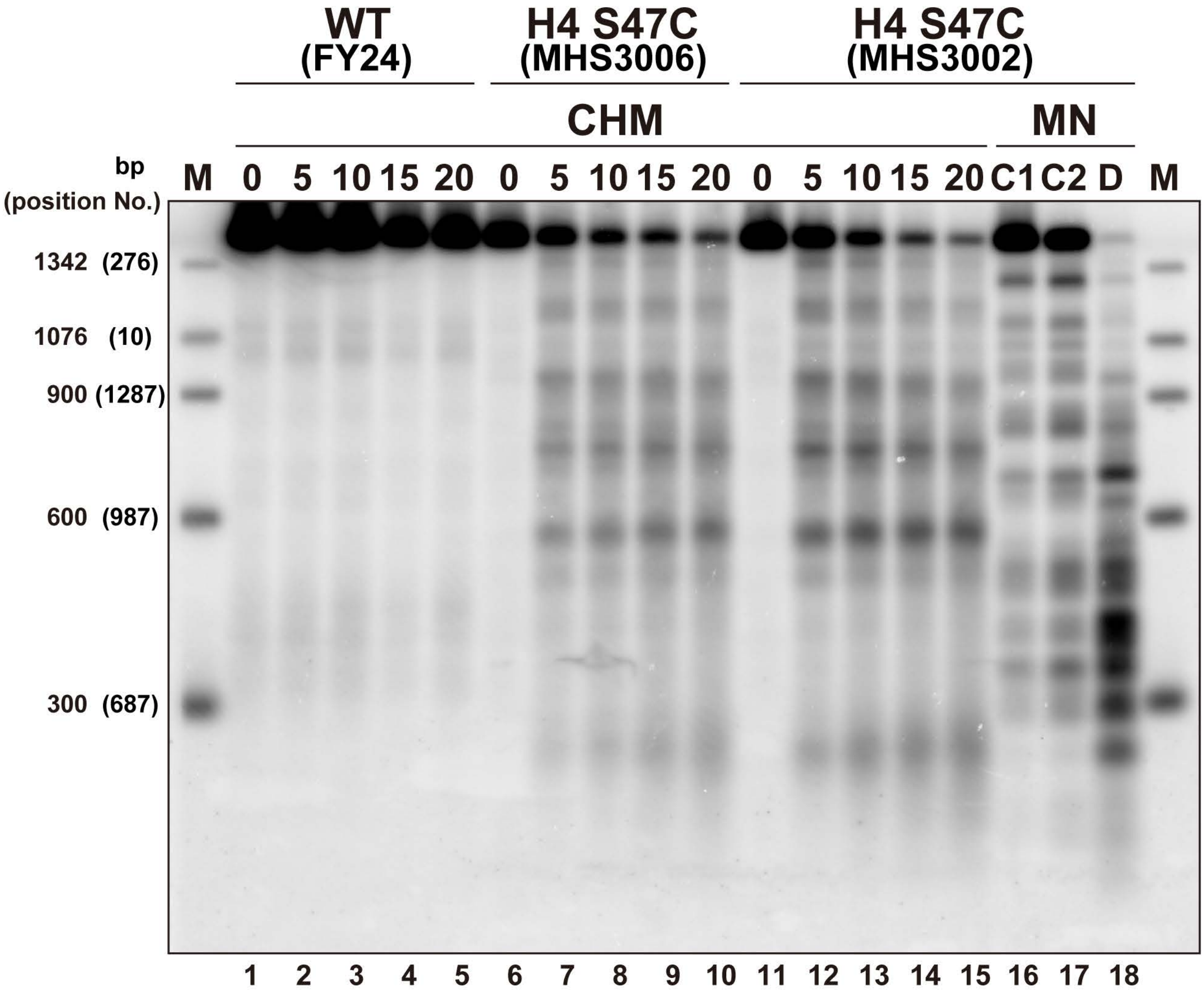

Fuse et al., Figure C

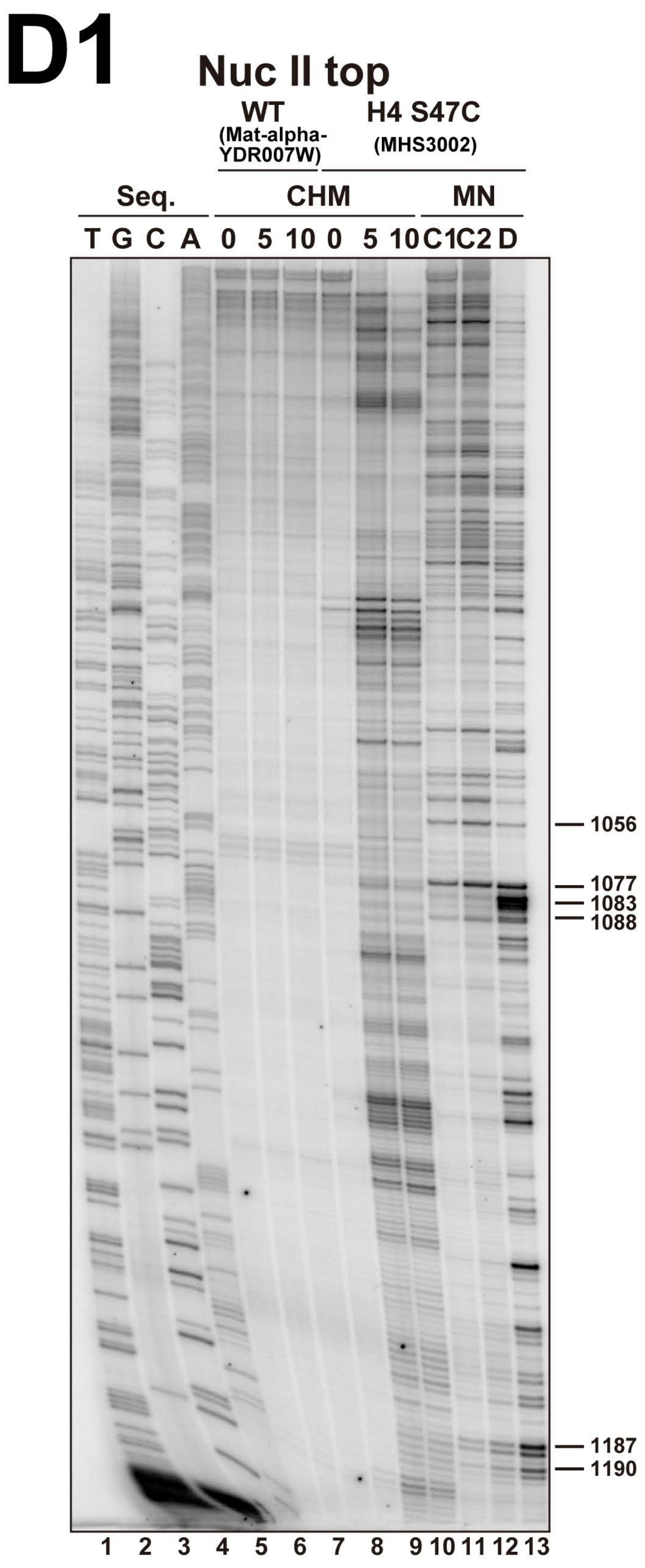

Fuse et al., Figure D1

D2 Nuc II bottom

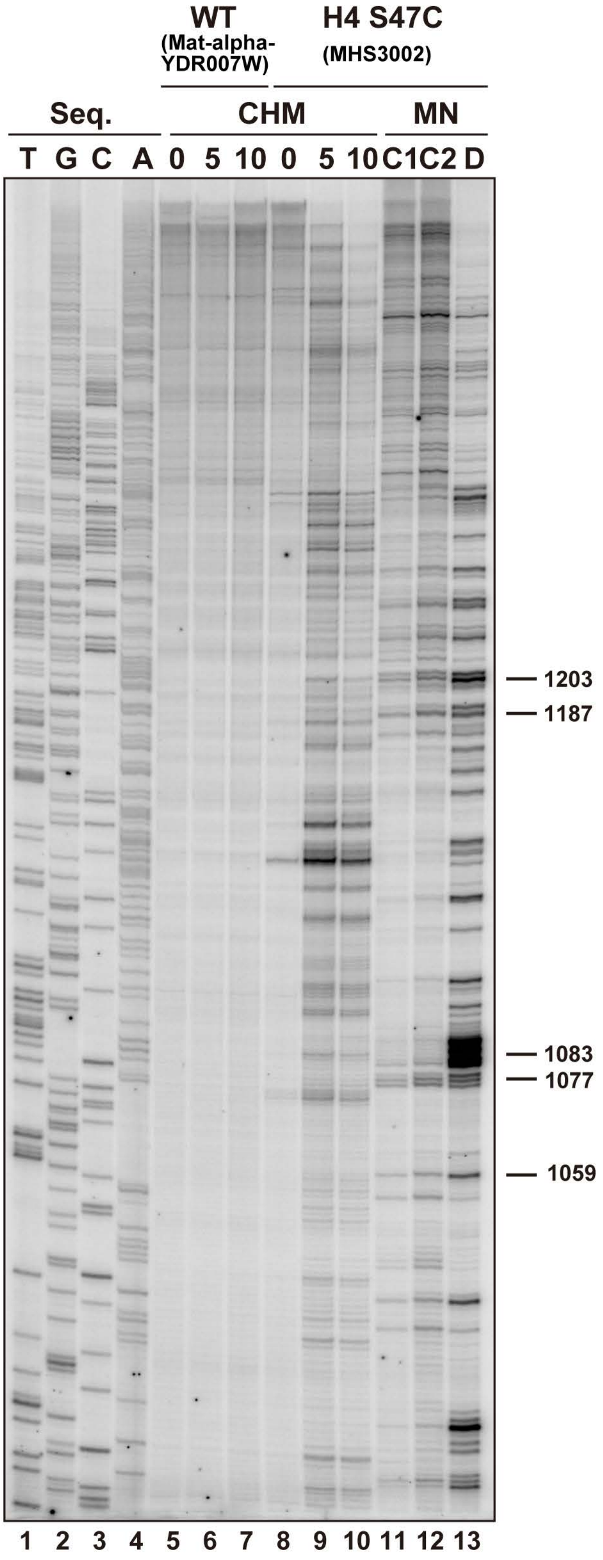

Fuse et al., Figure D2

D3

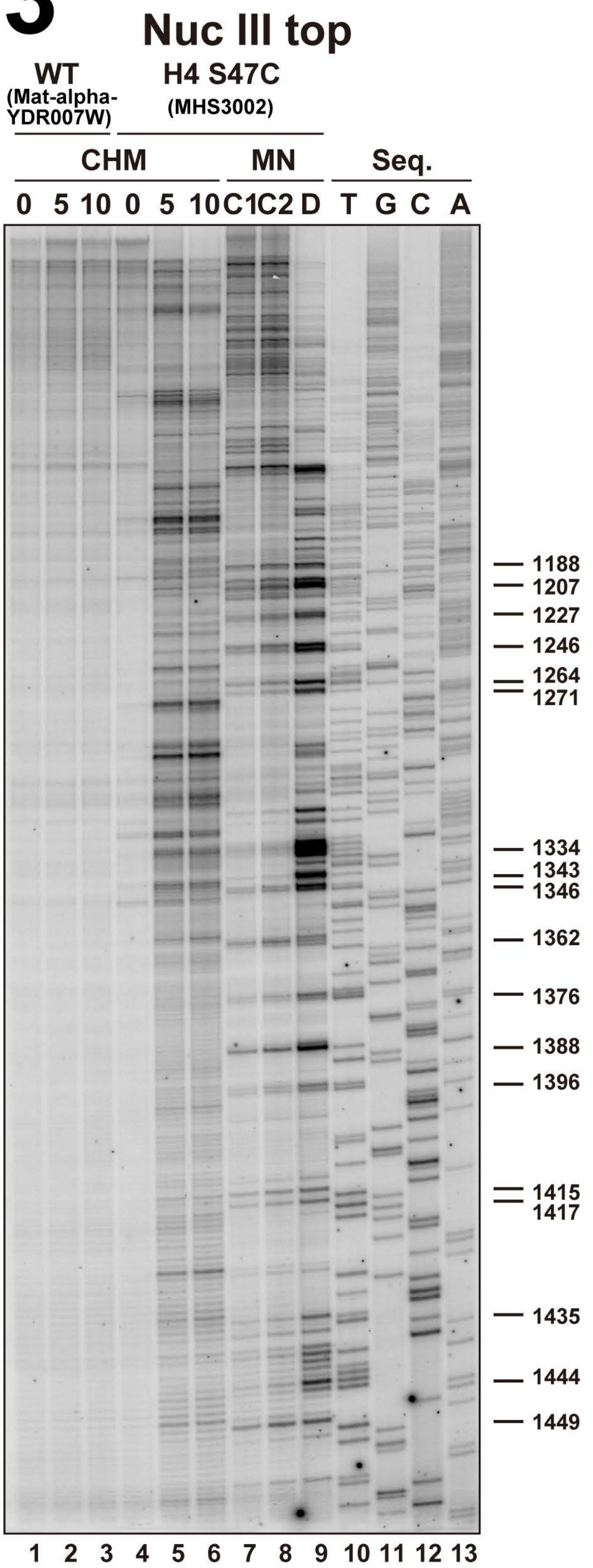

Fuse et al., Figure D3

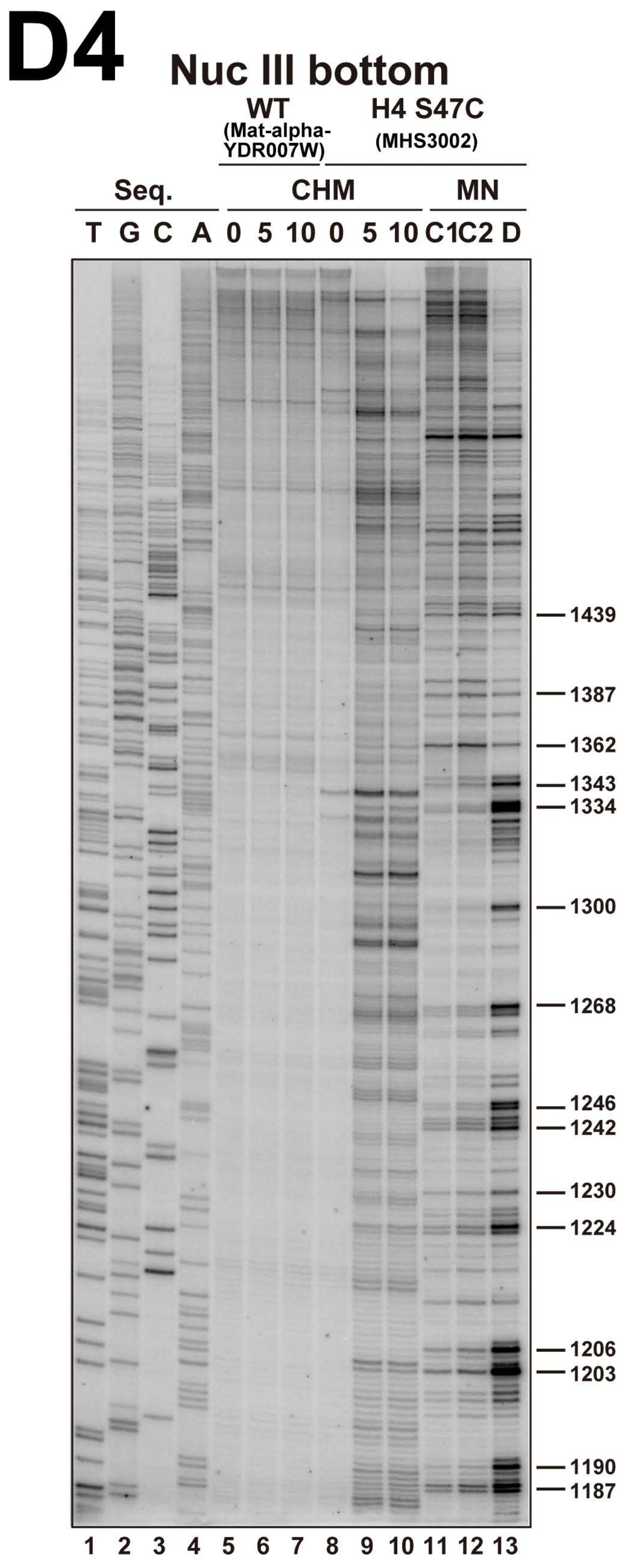

Fuse et al., Figure D4

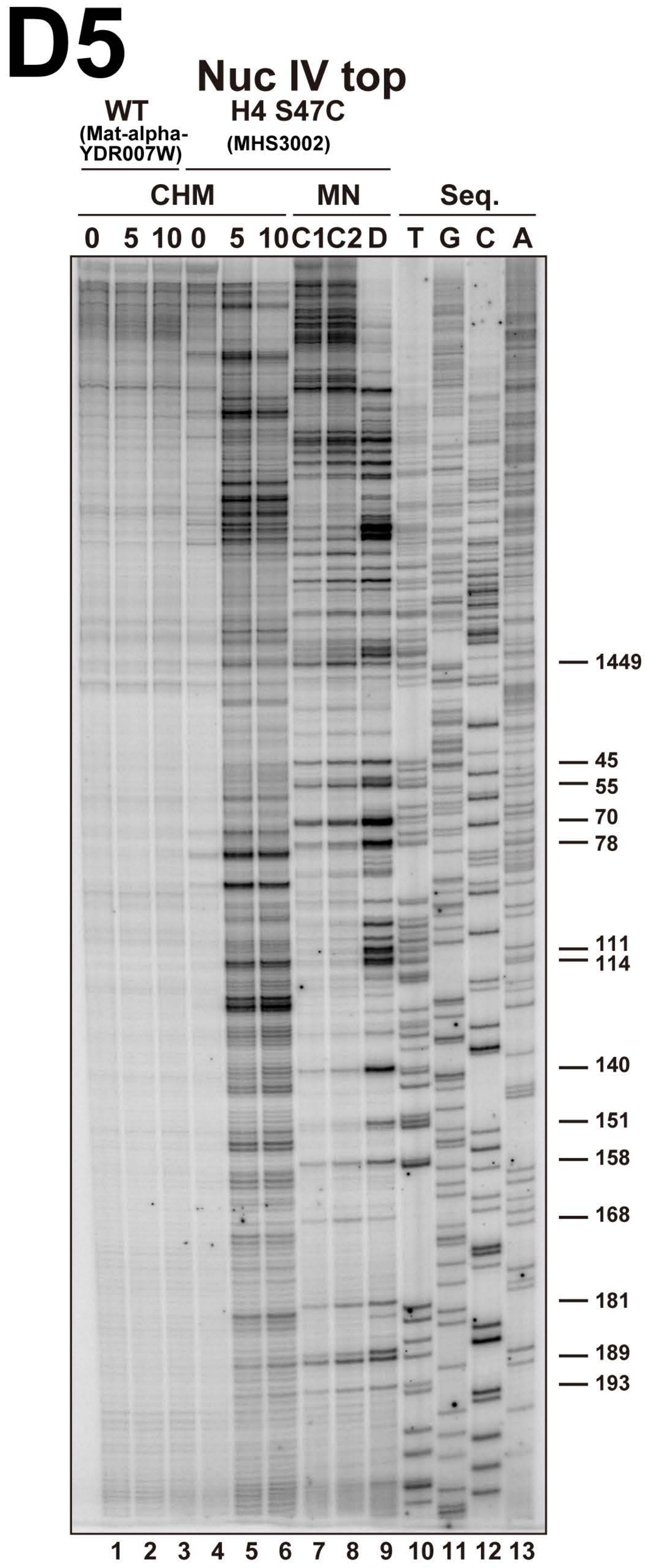

Fuse et al., Figure D5

D6 Nuc IV bottom

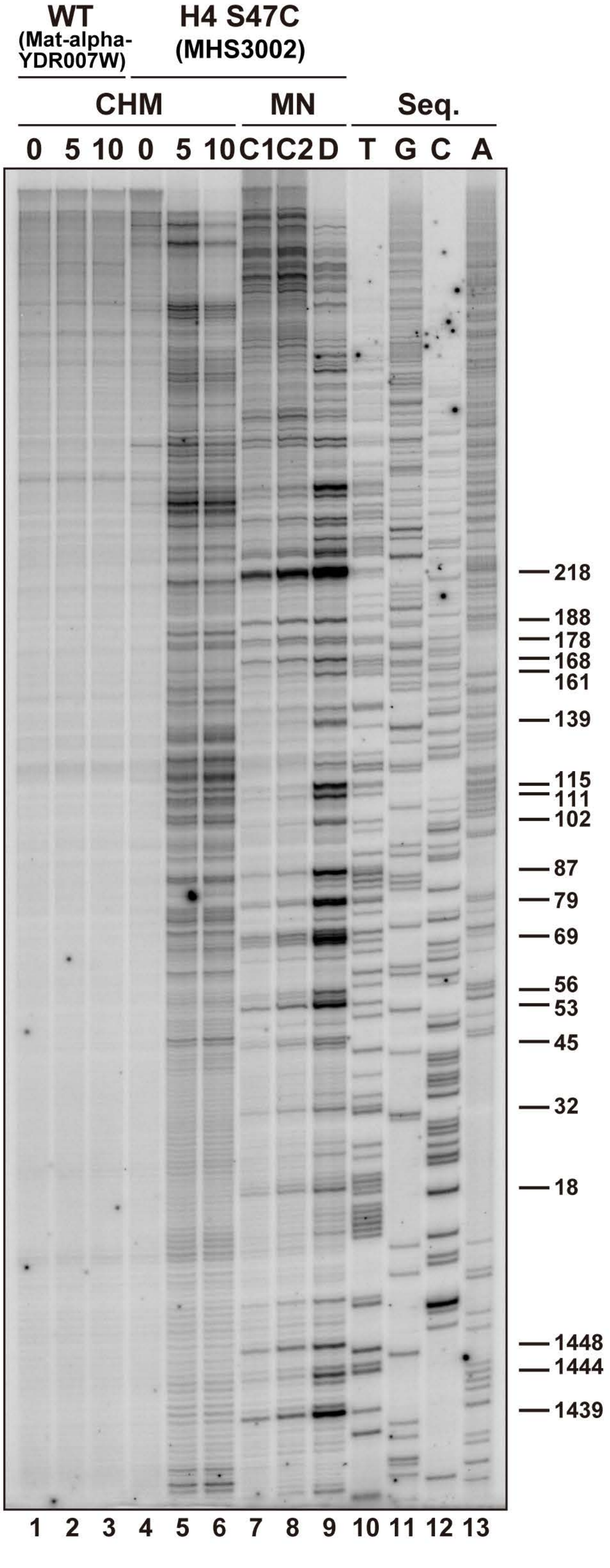

Fuse et al., Figure D6

E1

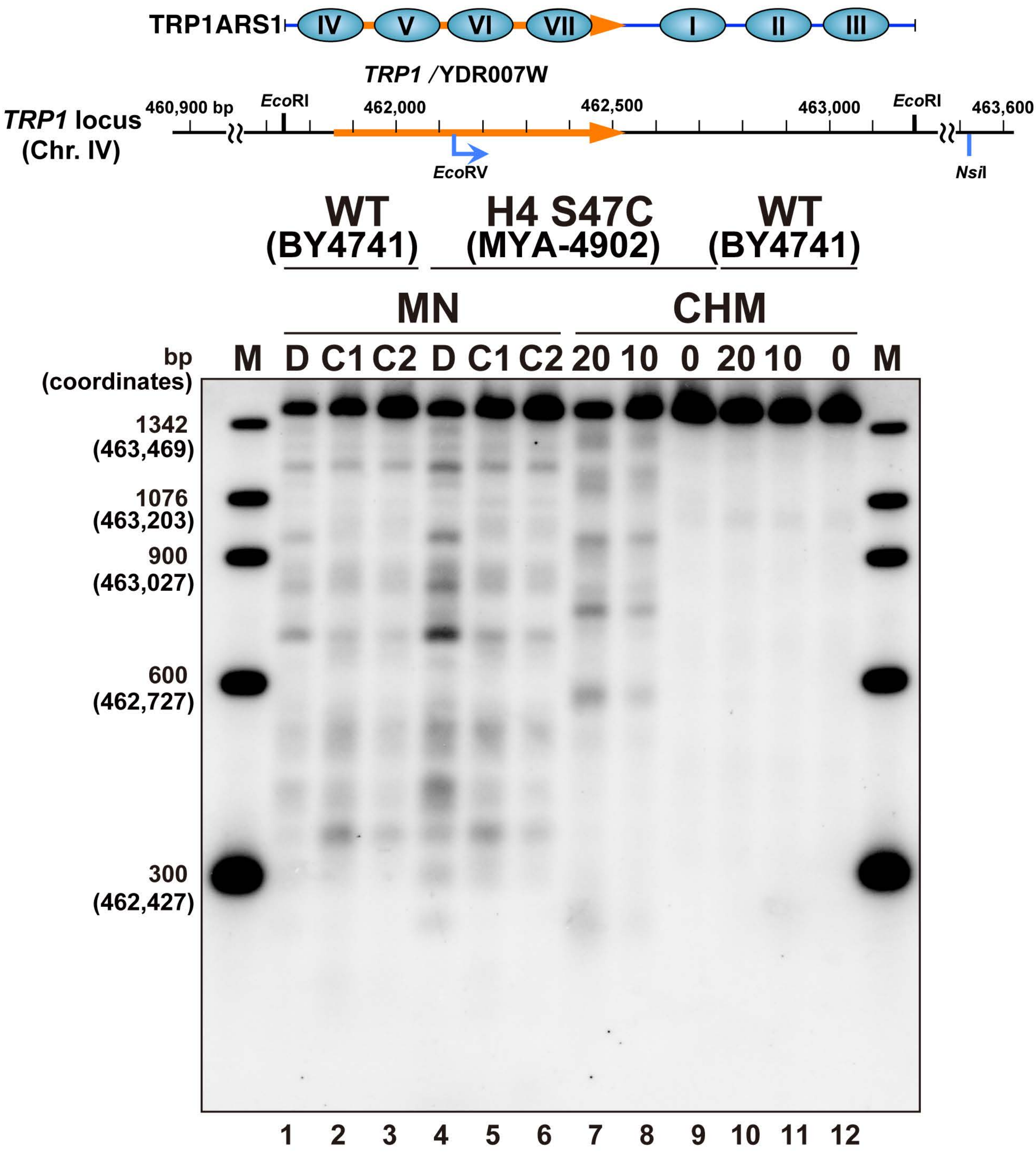
TRP1ARS1
IV
V
VI
VII
I
II
III
TRP1 / YDR007W
TRP1 locus
(Chr. IV)
460,900 bp
EcoRI
462,000
462,500
463,000
EcoRI
463,600
EcoRV
NsiI
WT
(BY4741)
H4 S47C
(MYA-4902)
WT
(BY4741)
MN
CHM
bp
(coordinates)
M
D
C1
C2
D
C1
C2
20
10
0
20
10
0
M
1342
(463,469)
1076
(463,203)
900
(463,027)
600
(462,727)
300
(462,427)
1
2
3
4
5
6
7
8
9
10
11
12

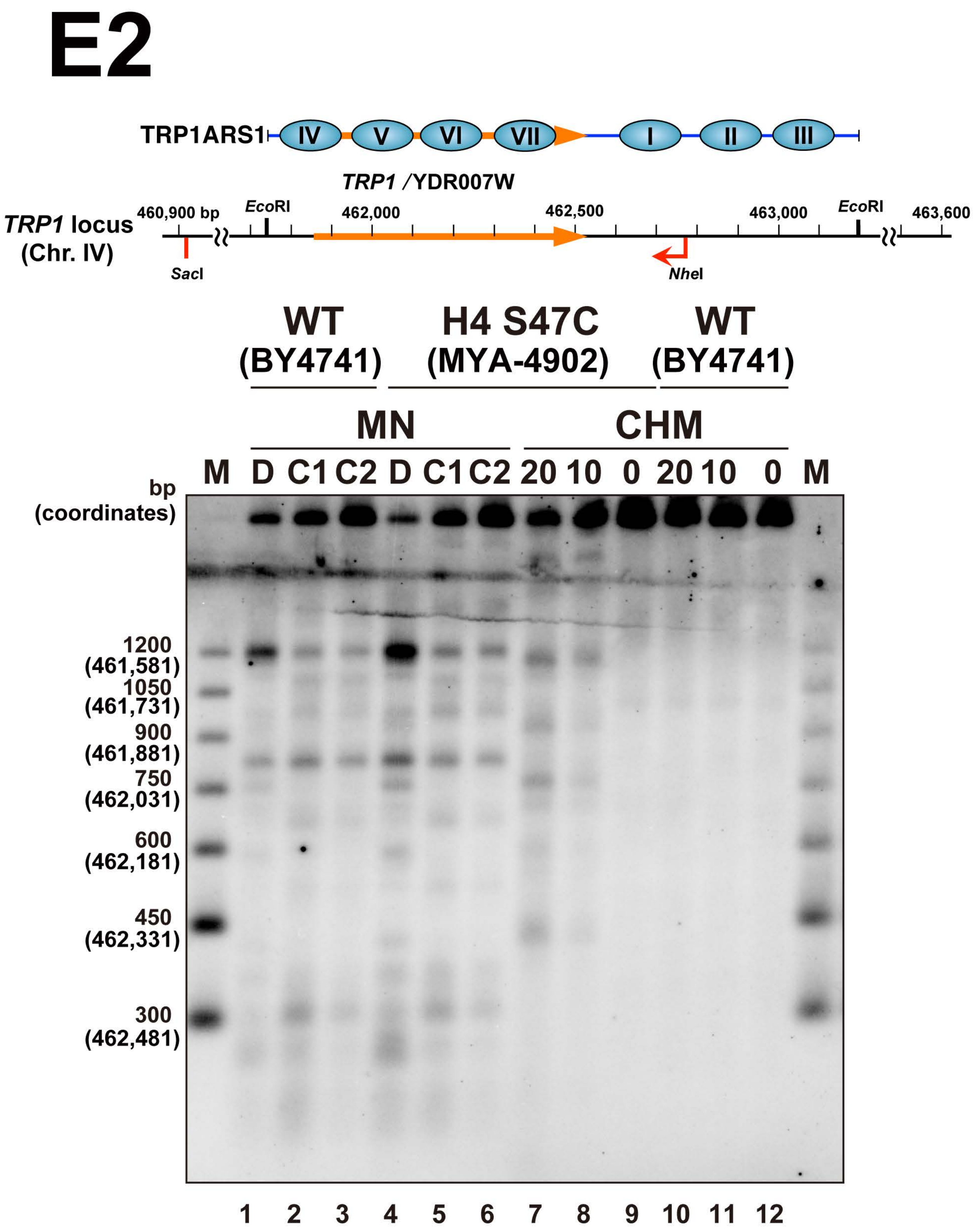
E2
TRP1ARS1
IV
V
VI
VII
I
II
III
TRP1 / YDR007W
TRP1 locus (Chr. IV)
460,900 bp
EcoRI
462,000
462,500
463,000
EcoRI
463,600
SacI
NheI
WT (BY4741)
H4 S47C (MYA-4902)
WT (BY4741)
MN
CHM
M D C1 C2 D C1 C2 20 10 0 20 10 0 M
bp (coordinates)
1200 (461,581)
1050 (461,731)
900 (461,881)
750 (462,031)
600 (462,181)
450 (462,331)
300 (462,481)
1 2 3 4 5 6 7 8 9 10 11 12

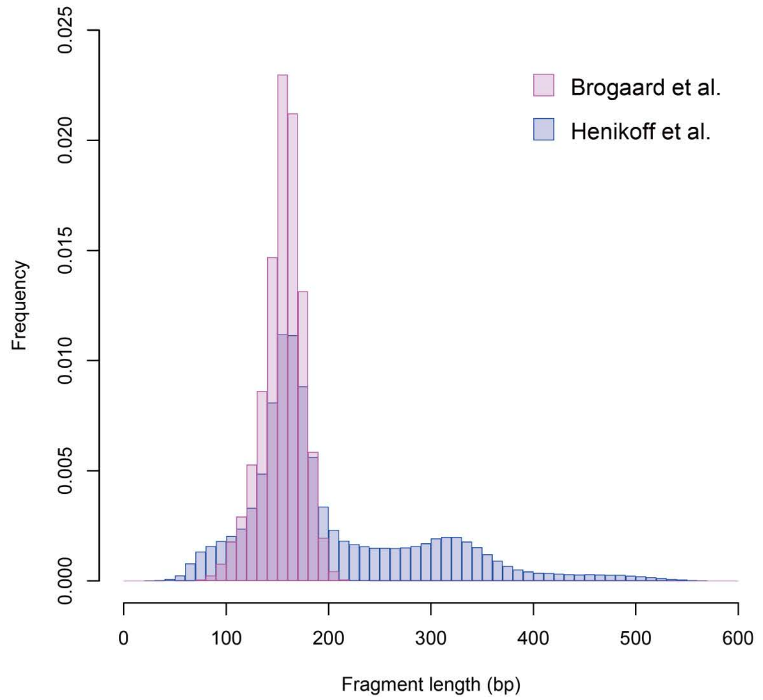

Fuse et al., Figure F
